# Cancer cell specific inhibition of Wnt/β-catenin signaling by forced intracellular acidification

**DOI:** 10.1038/s41421-018-0033-2

**Published:** 2018-07-03

**Authors:** Svitlana Melnik, Dmytro Dvornikov, Karin Müller-Decker, Sofia Depner, Peter Stannek, Michael Meister, Arne Warth, Michael Thomas, Tomas Muley, Angela Risch, Christoph Plass, Ursula Klingmüller, Christof Niehrs, Andrey Glinka

**Affiliations:** 1 Division of Epigenetics and Cancer Risks Factors, German Cancer Research Center, Heidelberg, D-69120 Germany; 2 DNA vectors, German Cancer Research Center, Heidelberg, D-69120 Germany; 3 Division of Systems Biology and Signal Transduction, German Cancer Research Center, Heidelberg, D-69120 Germany; 4 Translational Lung Research Center Heidelberg (TLRC), German Center for Lung Research (DZL), Heidelberg, Germany; 5 Tumor Models Unit, Center for Preclinical Research, German Cancer Research Center, Heidelberg, D-69120 Germany; 6 Division of Molecular Embryology, DKFZ-ZMBH Allianz, German Cancer Research Center, Heidelberg, D-69120 Germany; 7 Translational Research Unit, Thoraxklinik at University Hospital Heidelberg, Heidelberg, D-69126 Germany; 8 Institute of Pathology, Heidelberg University Hospital, Heidelberg, 69120 Germany; 9 Department of Molecular Biology, University of Salzburg, Salzburg, 5020 Austria; 10 Cancer Cluster Salzburg, Salzburg, 5020 Austria; 11 Institute of Molecular Biology (IMB), Mainz, 55128 Germany

## Abstract

Use of the diabetes type II drug Metformin is associated with a moderately lowered risk of cancer incidence in numerous tumor entities. Studying the molecular changes associated with the tumor-suppressive action of Metformin we found that the oncogene *SOX4*, which is upregulated in solid tumors and associated with poor prognosis, was induced by Wnt/β-catenin signaling and blocked by Metformin. Wnt signaling inhibition by Metformin was surprisingly specific for cancer cells. Unraveling the underlying specificity, we identified Metformin and other Mitochondrial Complex I (MCI) inhibitors as inducers of intracellular acidification in cancer cells. We demonstrated that acidification triggers the unfolded protein response to induce the global transcriptional repressor *DDIT3*, known to block Wnt signaling. Moreover, our results suggest that intracellular acidification universally inhibits Wnt signaling. Based on these findings, we combined MCI inhibitors with H^+^ ionophores, to escalate cancer cells into intracellular hyper-acidification and ATP depletion. This treatment lowered intracellular pH both in vitro and in a mouse xenograft tumor model, depleted cellular ATP, blocked Wnt signaling, downregulated *SOX4*, and strongly decreased stemness and viability of cancer cells. Importantly, the inhibition of Wnt signaling occurred downstream of β-catenin, encouraging applications in treatment of cancers caused by *APC* and *β-catenin* mutations.

## Introduction

Epidemiological studies have established that regular use of Metformin lowers the incidence risk for many cancer entities, including colorectal adenocarcinoma^1^. Also, Metformin has been shown to cooperate in elimination of cancer cells in combination with a number of other drugs^2,3^, as well as with radiotherapy^4,5^. Metformin represents a universal, but very weak, anticancer drug. Meta-analyses of multiple studies support its antitumor effect, affecting, for example, cancers of lung^6^, prostate^7^, and endometrium^8^. However, in some studies no improvement was observed, e.g., with non-small cell lung cancer (NSCLC)^9^.

The mechanism for Metformin’s anti-cancer specificity remains unclear. It has been proposed that the antidiabetic property of Metformin might also account for its anticancer effect. While previous studies have suggested that activation of AMPK (AMP-activated protein kinase) mediates the anticancer action of Metformin^10,11^, this has remained controversial^12,13^. In addition, Metformin can inhibit mitochondrial GPD2 (Glycerol-3-Phosphate Dehydrogenase 2)^14^. A characteristic feature of Metformin and other biguanidine-type drugs is their ability to reduce cellular ATP level by inhibition of mitochondrial complex I (MCI) accompanied by compensatory increase rate of glycolysis in sensitive cells^15^. In general, MCI inhibitors are known for their anticancer properties^16–18^. A moderate inhibition of MCI with therapeutical doses of MCI inhibitors causes no side effects in routine medical practice (Metformin, Phenformin, and Papaverine). However, a disadvantage of Metformin as an anticancer drug is a necessity to apply it at very high concentrations in in vitro experiments to achieve substantial effects. To reach similar outcome in cancer patients, the drug has to be applied at doses that might trigger lactic acidosis as a side-effect. Insights into Metformin’s anticancer mechanism could help to suggest more effective drugs with similar but enhanced properties.

Since Metformin affects tumor cells from multiple tissue entities, this suggests that there are some underlying common molecular markers. Evaluation of these markers would help monitoring molecular changes caused by Metformin. The most pronounced anticancer effects for Metformin have been reported for colorectal adenocarcinoma cases^1^. It is well-established that in many instances colorectal cancer is caused by aberrant Wnt signaling^19,20^. At the same time, *SOX4* (SRY (Sex Determining Region Y)-box 4), a transcription factor and oncogene expressed in many types of tumors^21,22^, has been found to be a prognostic marker of poor outcome for colon cancer patients^23^. These observations point to a probable link between Metformin, Wnt signaling and SOX4. High expression levels of *SOX4* correlate with cancer patients mortality rates, regardless of other clinical parameters^21^. Conversely, it has been demonstrated that knockdown of the *SOX4* gene in xenograft model suppresses tumor growth^24^. Normal *SOX4* expression is limited to embryonic cells and some adult tissues such as pancreas, intestine, and skin. It is also expressed in a number of human non-cancer cell lines of embryonic origin^25^. SOX4 expression is linked to cell migration, proliferation, Epithelial-to-Mesenchymal transition (EMT) and metastasis formation^26^. Thus, *SOX4* would be a candidate for a universal oncogene that is independent of a tumor entity, and at the same time is expressed in non-cancer cells of embryonic origin. These two important features could be used to assess both specificity and efficiency of tested cancer-suppressing treatments.

Upregulation of Wnt signaling is a strong cancer-driving force for multiple types of malignancies^19^, and in particular, is a primary cause of colon cancer^20^. Predominant reasons for such Wnt signaling upregulation are loss-of-function mutations for *APC*, which promotes β-catenin degradation. As a result, β-catenin protein accumulates and, upon binding LEF/TCF transcription factors and co-activators of transcription CBP/p300, forms an ‘activator complex’. This complex binds to LEF/TCF binding sites at promoters of Wnt target genes. Abnormal accumulation of β-catenin therefore causes overexpression of specific Wnt target genes, including *AXIN2*, and a number of potential oncogenes^27^. In addition, a variety of stabilizing mutations in *β-catenin* gene cause similar effects as *APC* mutations. *β-catenin* and *APC* mutations account for 95% incidences of colorectal cancer. Mutations resulting in β-catenin accumulation are not limited to colon cancer, and often found in tumors of other origin: liver (hepatocellular carcinoma^28^), kidney^29^, ovary^30^, prostate^31^, brain (medulloblastoma^32^), endometrial cancer^33^ and thyroid gland^34^. In addition, Wnt signaling is a major positive contributor in multiple cancer stem cells functions^27,35^ and also is a driving force of lung adenocarcinoma^36^.

Multiple attempts have been made to develop drugs inhibiting Wnt signaling (reviewed by Novellasdemunt et al.,^20^). Only a few of the found drugs could target β-catenin/TCF interactions, to block Wnt signaling at the level of β-catenin^37^. A main pitfall of these drugs, however, is the absence of specificity towards cancer cells, and accompanying side effects.

In this study, we addressed the mechanism of the universal anticancer properties of Metformin and discovered its ability to block Wnt signaling specifically in cancer cells. We applied these findings to develop a new cancer cell specific strategy for Wnt/β-catenin signaling inhibition that exploits a characteristic feature of cancer cell metabolism, - the Warburg effect^38^. We found that this strategy resulted in consequent cancer cells elimination without causing any significant effects in non-cancer cells.

## Results

### Metformin inhibits Wnt/β-catenin signaling

**Fig. 1:**
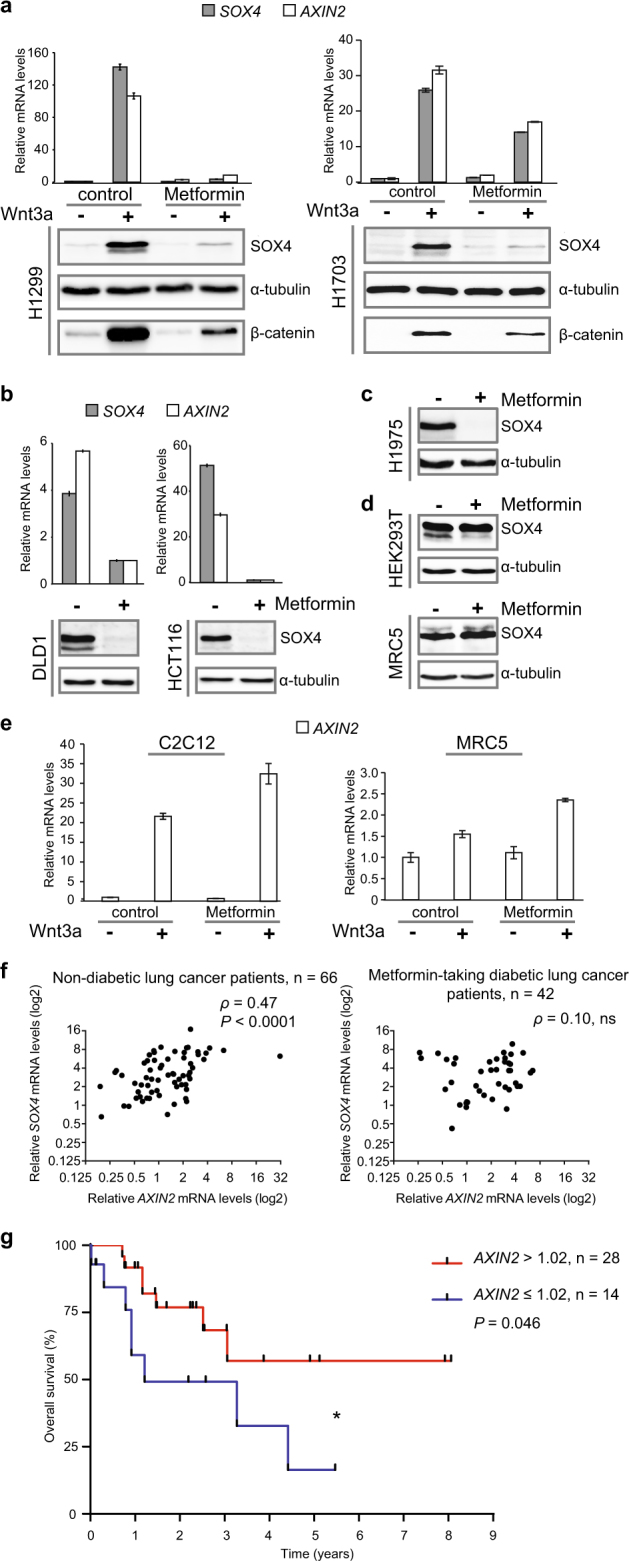
Metformin inhibits Wnt signaling. **a**–**e** Levels of the indicated mRNAs and proteins were monitored in the indicated cell lines by qRT-PCR or WB, respectively. Error bars represent mean values ± SD. **f** Non-linear Spearman’s rank correlation analysis of *SOX4* mRNA expression levels with Wnt signaling (*AXIN2*) in tumor tissue samples from lung cancer patients, non-diabetic or Metformin-taking diabetic. *ρ* Spearman’s rank coefficient for non-linear correlation; *P* *<* 0.0001 (****); ns -  not significant. **g** Kaplan–Meier overall survival curves of the Metformin-taking diabetic lung cancer patient cohort stratified by *AXIN2* expression levels (mRNA threshold expression level = 1.02, log-rank test, *P* *=* 0.046 (*)).

Wnt signaling can be induced in cultured cells by applying Wnt3a protein and monitored either by measuring β-catenin protein stabilization or induction of its immediate downstream target gene *AXIN2*. We found that Metformin inhibits Wnt signaling as monitored by *AXIN2* expression, but only moderately affects levels of β-catenin protein accumulation (Fig. 1a). Moreover, we found that the expression of an oncogene *SOX4*, which we identified to be Wnt3a-inducible, and with similar kinetics of mRNA and protein levels induction to the Wnt target gene *AXIN2* (Supplementary Fig. S1a-b), was also inhibited by Metformin (Fig. 1a-c). Treatment with *siβ-catenin* RNA prevented *SOX4* induction upon Wnt3a treatment, and conversely, *SOX4* was induced by *β-catenin* overexpression (Supplementary Fig. S1c-d). However, in cancer cell lines DLD1, HCT116, and H1975, *siβ-catenin* did not affect *SOX4* expression (Supplementary Fig. S1e), indicating that *SOX4* expression does not universally require Wnt signaling. Yet, in these cell lines, Metformin still blocked SOX4 (Fig. 1b-c), suggesting that Metformin can affect SOX4 expression also independently of Wnt signaling. Interestingly, Metformin had no inhibiting effect on SOX4 and *AXIN2* levels in non-cancer cell lines (HEK293T, MRC5, and C2C12, Fig. 1d-e). We conclude that expression of SOX4 is Wnt-inducible and sensitive to Metformin preferentially in cancer cells.

Next, we addressed the question whether regular intake of Metformin by diabetic cancer patients might have an impact on expression of *SOX4* and other Wnt target genes in their tumor tissue. For this purpose, we analyzed a lung cancer patient cohort, as it has been shown that lung cancer is associated with aberrant Wnt signaling^39^. No significant changes in mean values of *SOX4* and *AXIN2* between non-diabetic and diabetic Metformin-taking lung cancer patients were detected (Supplementary Table S1). However, there was a significant correlation between *SOX4* and *AXIN2* mRNA levels in tumor tissues in the group of non-diabetic lung cancer patients (Spearman’s rank correlation coefficient *ρ* *=* 0.47, *P* *<* 0.0001). This correlation was lost in tumor samples of the diabetic Metformin-taking lung cancer cohort (Fig. 1f). Since SOX4 expression and EMT are common mechanisms underlying metastasis^26^, we tested whether levels of EMT markers in lung tumor samples were also affected by Metformin intake. Indeed, there was a significant correlation between levels of *AXIN2* and the EMT markers *VIM* (*Vimentin*) and *ZEB1 (Zinc Finger E-Box Binding Homeobox 1*), which again was lost in tumor samples from Metformin-taking lung cancer patients (Supplementary Fig. S2). Interestingly, within the Metformin-taking cohort, higher initial levels of *AXIN2* mRNA (‘high Wnt group’) correlated with prolonged survival (*P* *=* 0.046) (Fig. 1g). We speculate that the high *AXIN2* group represents Wnt-addicted tumors, suggesting that patients with higher Wnt signaling might particularly benefit from Metformin treatment.

### Metformin treatment abolishes TCF4/β-catenin/CBP-p300 activation complex formation

**Fig. 2:**
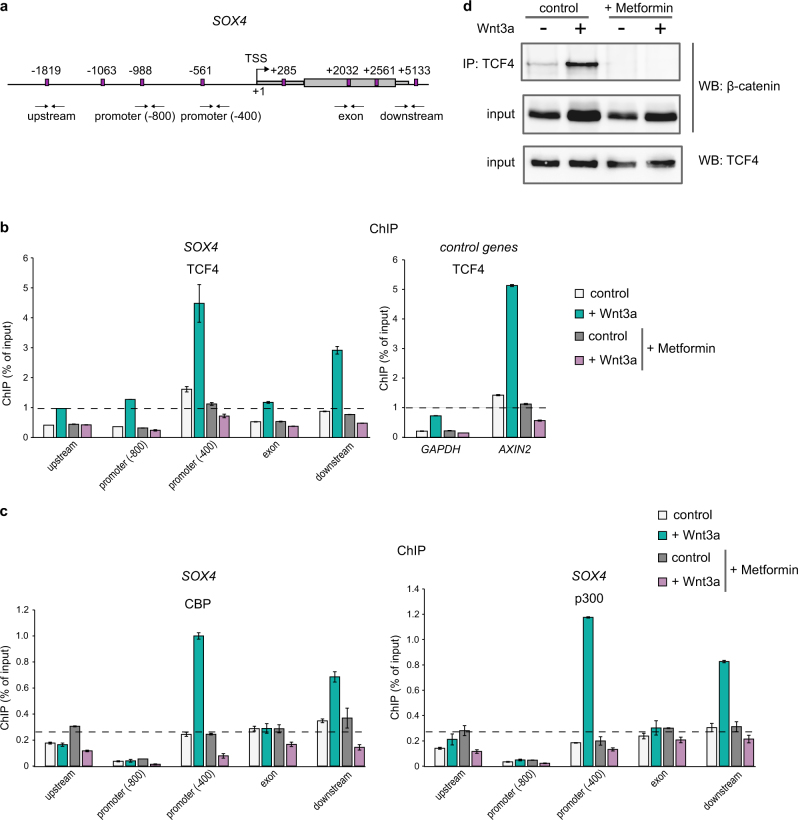
Metformin treatment abolishes TCF4/β-catenin/CBP-p300 activation complex formation. **a** Scheme of human *SOX4* gene locus. Putative LEF/TCF binding sites marked with magenta boxes, and their positions in relation to the transcription start site (TSS, top arrow) are indicated. *SOX4* gene consisting of a single exon is marked with a gray box; left and right thinner gray boxes mark positions of the 5’- and 3’-UTR regions, respectively. Small double arrows below the locus indicate positions of primer pairs used for ChIP analysis within the locus. **b**–**c** ChIP assay done in H1299 cells treated with Wnt3a and Metformin, as indicated. After DNA recovery, binding efficiencies for TCF4 (**b**) or CBP and p300 (**c**) antibodies at 5 genomic locations spanning upstream, promoter (−800 and −400), exon and downstream regions of *SOX4* gene, as well as a promoter of *GAPDH* and a known site for TCF4 binding at *AXIN2* (**b**), - negative and positive TCF4 binding control genes, respectively, were measured by qPCR. Values were normalized to the total DNA input. Level of unspecific binding determined with normal IgG ChIP reactions are indicated with dashed black line. Error bars correspond to mean values ± SD. **d** Co-immunoprecipitation (Co-IP) assay done in H1299 treated as in (**b**–**c**). After lysis, cells were subjected to immunoprecipitation with TCF4 antibody bound to agarose beads, and immunocomplexes were tested by WB with β-catenin antibody

Next, we addressed whether TCF4 (T-cell Factor 4, a member of LEF/TCF protein family) remains bound to the *SOX4* promoter upon Metformin treatment. We inspected the *SOX4* gene locus and found four putative sites for LEF/TCF transcription factors binding (containing the consensus sequence CAAAG^40^) within the *SOX4* promoter region. Another four putative TCF4 binding sites were found in close proximity downstream in the gene body (Fig. 2a). By chromatin immunoprecipitation assay (ChIP) we found that, indeed, the proximal promoter of *SOX4* gene (at position -400 from transcription start site (TSS)) strongly binds TCF4 upon treatment with Wnt3a. Similar binding was detected to *AXIN2*, a positive control gene for TCF4 binding^41^, in contrast to a negative control, *GAPDH* gene. However, Metformin treatment completely abolished TCF4 binding, for both *SOX4* and *AXIN2* genes (Fig. 2b).

We then analyzed whether the mechanisms that have been proposed to underlie the antidiabetic effect of Metformin might also account for its anticancer properties. While previous studies have suggested that activation of AMPK mediates the anticancer action of Metformin^10,11^, this remains controversial^12,13^. In addition, Metformin has been shown to inhibit mitochondrial GPD2^14^. In our experiments, however, neither *siGPD2*, nor *siAMPK* interfered with the Metformin'sinhibiting effect on SOX4 expression (Supplementary Fig. S3a-b). Metformin has also been shown to reduce gene activation in gluconeogenesis by promoting phosphorylation of the histone acetyltransferase (HAT) CBP, but not of the related HAT p300^42^. CBP (also known as CREB-binding protein or CREBP) and p300 (p300 known as EP300 or E1A binding protein p300), share 63% homology in their sequences and structures and are involved in multiple cellular processes functioning as transcriptional co-factors and histone acetyltransferases^43^. It has been shown that Metformin treatment causes phosphorylation of murine CBP at Ser436 blocking its binding to promoters of gluconeogenesis enzymes genes, whereas p300, which has Ala instead of Ser at this position, retains its activity^42^. We assayed whether Metformin utilizes its effect on SOX4 in similar fashion and exclusively prevents CBP binding at the promoter of *SOX4* gene over p300. We found that Metformin treatment abrogated binding to the *SOX4* promoter of both, CBP and p300 (Fig. 2c), arguing against CBP as Metformin´s specific target in *SOX4* inhibition. Moreover, by Co-immunoprecipitation assay (Co-IP), we found that TCF4/β-catenin complex formation is affected by Metformin treatment in general, as this interaction induced by Wnt3a, was disrupted by the drug (Fig. 2d).

### DDIT3 is one of the key mediators of Wnt signaling inhibition

**Fig. 3:**
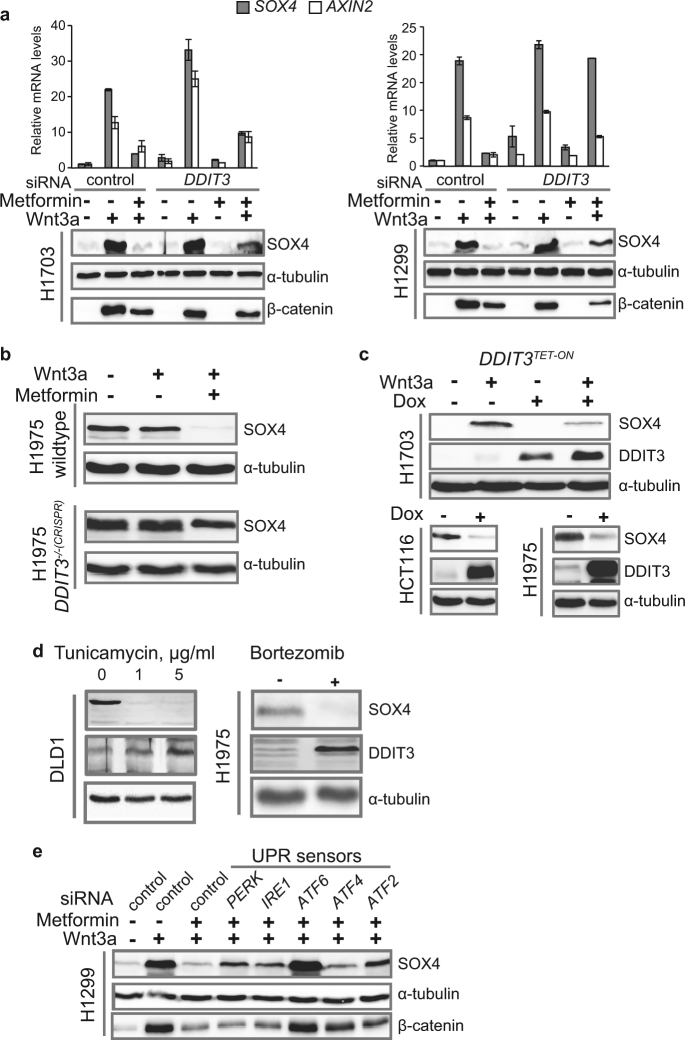
DDIT3 is a key mediator of SOX4 repression and Wnt signaling inhibition. **a**–**e** Levels of the indicated mRNA and proteins in the indicated cell lines were monitored by qRT-PCT or WB, respectively. **a** SOX4 rescue by *DDIT3* loss-of-function. Error bars represent mean values ± SD. **b** SOX4 rescue in cells with DDIT3 knockout (*DDIT3*^-/-*(CRISPR)*^) in comparison to wildtype. Metformin treatment  -  35 h. **c** SOX4 inhibition caused by overexpression of *DDIT3* in *DDIT3*^*TET-ON*^ cells induced by Doxycycline (Dox). **d** DDIT3 induction and removal of SOX4 protein caused by ER drugs, Tunicamycin and Bortezomib. **e** SOX4 rescue caused by siRNA-mediated knockdown of the indicated members of the UPR.

The inhibition of TCF4 binding to target gene promoters accompanied by removal of CBP/p300 suggested that Metformin might induce some transcriptional repressor. We focused on the stress response transcription factor DDIT3 (DNA damage-inducible transcript 3, CHOP, GADD153), since it is known to be Metformin-inducible^44^ and inhibiting Wnt signaling *via* LEF/TCF binding^45^. We found that in concentrations where Metformin induces DDIT3 protein (Supplementary Fig. S4a-c), it repressed Wnt signaling (Supplementary Fig. S4d). In a loss-of-function approach, *siDDIT3* blocked the ability of Metformin to inhibit *SOX4* induction by Wnt3a (Fig. 3a and Supplementary Fig. S5a). Using a *DDIT3*-mutant H1975 cell line created by CRISPR/Cas9 gene editing, we found that *DDIT3* knockout completely abrogated SOX4 inhibition by Metformin (Fig. 3b and Supplementary Fig. S4e). Conversely, *DDIT3* overexpression in three different cell lines using the Tet-on system inhibited SOX4 expression, thus mimicking the effect of Metformin (Fig. 3c). This suggests that DDIT3 induction directly mediates SOX4 inhibition by Metformin. Indeed, the DDIT3 inducers, Tunicamycin^46^ and Bortezomib^47^, also blocked SOX4 expression (Fig. 3d and Supplementary Fig. S6a-b). Additionally, consistent with inhibition of *SOX4*, a gene promoting metastasis and cell invasion^22^, both Metformin and Bortezomib significantly reduced the invasion characteristics of cancer cells induced with Wnt3a treatment (Supplementary Fig. S6c).

Both Tunicamycin and Bortezomib induce endoplasmic reticulum (ER) stress, and DDIT3 is a component mediating the Unfolded Protein Response (UPR)^48^. We examined whether other key components of the UPR might also mediate Metformin’s action. Indeed, targeting the other UPR components (PERK, IRE1, ATF2, ATF4, and ATF6) by siRNA also repressed Metformin´s ability to block SOX4 induction by Wnt3a, with ATF6 being the most prominent (Fig. 3e and Supplementary Fig. S5b).

### Metformin and MCI inhibitors cause intracellular acidification and induce the UPR

**Fig. 4:**
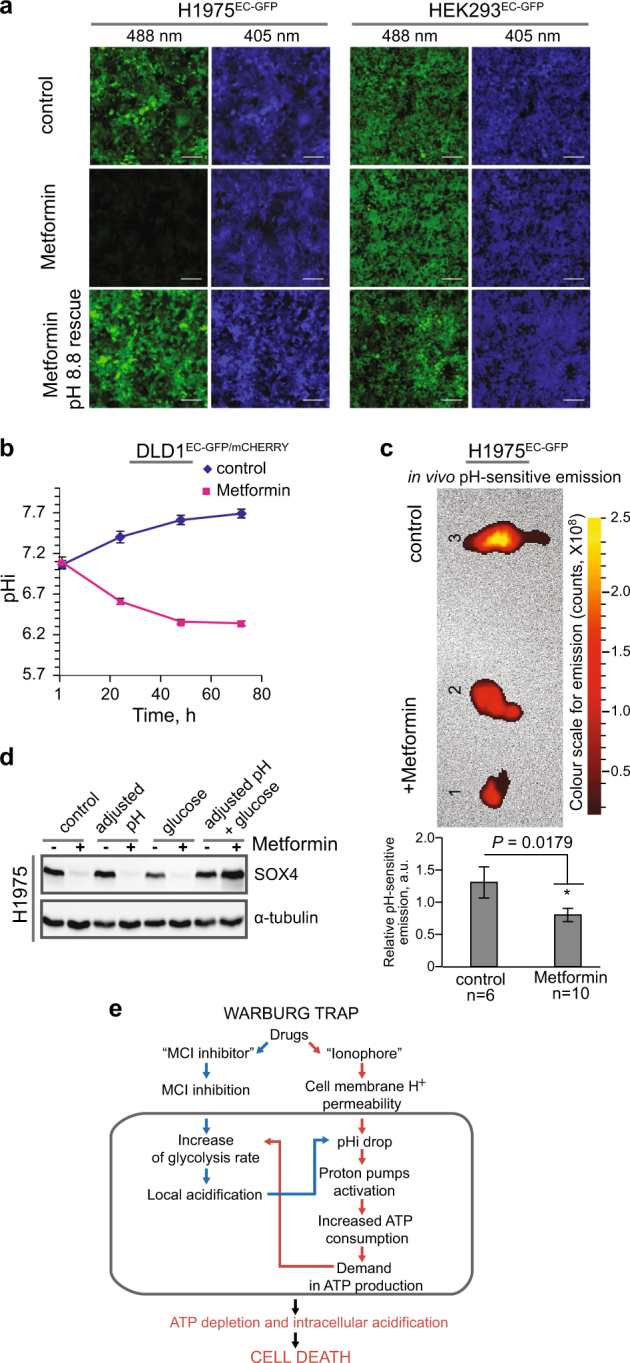
MCI inhibitors cause intracellular acidification. **a** Intracellular acidification monitored by live imaging in the indicated cell lines upon treatment with Metformin (48 h). After imaging, cells were transferred to media with pH 8.8 supplemented with 5 µM Nigericin, for 0.5 h, before re-imaging (Metformin pH 8.8 rescue). Scale bar, 150 µm. **b** Intracellular acidification time-course caused by Metformin treatment in DLD1^EC-GFP/mCHERRY^ cells. **c** In vivo intracellular acidification caused by Metformin monitored in mouse tumor xenografts induced with H1975^EC-GFP^ cells. Top: representative *ex vivo* xenograft images: tumors 1, 2—Metformin treated, tumor 3—control. Bottom: live animals pHi measurements, *P* *=* 0.0179 (*); n number of animals. **d** SOX4 evaluation by WB after pH adjustment to 7.4 and 4 mM glucose replenishment of culture media upon treatment H1975 cells with Metformin. **e** Warburg Trap principle scheme (**b–c**): Error bars correspond to mean values ± SEM

How does Metformin induce the UPR? We and others^49^ observed that culture media from cells under Metformin treatment tended to acidify. Hence, we hypothesized that Metformin treatment might also disturb cells’ ability to maintain the intracellular pH (pHi), thereby inducing ER stress and the UPR. It is known that the extracellular pH (pHe) in tumor microenvironment is reduced to as low as ~pH 5.5, and that acidosis is an important stress factor and selection force for cancer cell somatic evolution^50^. To test whether Metformin lowers the intracellular pH, we generated a stable H1975 lung cancer and HEK293 control cell line expressing a pH-sensitive variant of GFP (EC-GFP) (H1975^EC-GFP^ and HEK293^EC-GFP^), whose fluorescence is extinguished at low pH when excited at 488 nm, but not at 405 nm (‘E_488_’ vs. ‘E_405_’)^51^. Metformin treatment strongly reduced emission at E_488_, but not at E_405_, indicating lowering of pHi, and this was specific for the H1975^EC-GFP^ lung cancer cell line (Fig. 4a). We also established a human colon cancer cell line DLD1 expressing a combination of pH sensitive EC-GFP, as a pHi sensor, along with a pH-insensitive protein mCherry, as a control fluorescent protein (DLD1^EC-GFP/mCHERRY^). Using this reporter cell line, we analyzed the pHi dynamics with and without Metformin treatment. Cells were placed in slightly acidic medium (pH 6.5) mimicking acidified tumor microenvironment. Without Metformin, cancer cells predictably increased the pHi to compensate lower pHe (Fig. 4b, control)^52^, however, in the presence of Metformin the pHi was significantly decreased.

Next, we assayed the pHi changes in tumor tissue derived from human lung cancer H1975^EC-GFP^ cells xenotransplanted in nude mice that were given Metformin orally, once the tumor growth was detected. A week later after the treatment had started, mice were subjected to live imaging, and pH sensitive fluorescence of tumor cells was monitored, first in vivo (Fig. 4c, bottom) and then ex vivo, immediately after tumor excision (Fig. 4c, top). We detected, both in vivo and ex vivo, a significant (*P* = 0.0179) pHi drop in tumor tissues of tested animals that received Metformin compared to the control mice. We conclude that Metformin significantly decreases the intracellular pH in cancer cells, both in vitro and in vivo.

How does the treatment with Metformin affect the pHi? Metformin inhibits Mitochondrial Complex I (MCI) ^4^ and hence ATP production, leading to compensatory increase in glycolysis and acidification due to lactic acid production. We tested the effect on pHi of other known MCI inhibitors and found that, similar to Metformin, all caused a pHi drop (Supplementary Fig. S7a-b). However, this was not the case with an inhibitor of Mitochondrial complex II (Supplementary Fig. S7c), which produces less energy than complex I. Of note, MCI inhibitors are known for their anticancer properties^16–18^.

A common feature of cancer cells is a reversed pH gradient, i.e., a higher pHi and a lower pHe than in normal cells because their pHi homeostasis relies on extrusion of H^+^ by ATP-consuming proton pumps^53,54^. In case of an ATP deficit, proton pumps function is impaired leading to intracellular acidification. This scenario predicts that cancer cells might overcome Metformin inhibition when extra glucose is provided to support glycolysis as an alternative pathway for ATP production. Indeed, cell culture medium pH-adjustment combined with elevated glucose completely rescued SOX4 suppression by Metformin (Fig. 4d). This result is consistent with the earlier observation that cancer cells are more sensitive to Metformin at lowered glucose levels^49^.

To address a question whether the intracellular acidification alone would block Wnt signaling and cause similar effects as MCI inhibitors, we tested drugs thought to decrease pHi by affecting monocarboxylate transporters (MCT) and carbonic anhydrases (CA)^54^. We found that a broad MCT inhibitor CHC (2-Cyano-3-(4-hydroxyphenyl)-2-propenoic acid) was able to decrease pHi (Supplementary Fig. S8a), and this correlated with its Wnt signaling inhibition ability (Supplementary Fig. S8b). However, specific MCT-1 (AZD3965) or CA inhibitors (Azetazolamide) were not able to drop the pHi or inhibit Wnt signaling (Supplementary Fig. S8). We therefore conclude that any treatment causing pHi drop would inhibit Wnt signaling.

### MCI inhibitor and ionophore drug combinations inhibit Wnt signaling and cell viability

We reasoned that combined treatment with MCI inhibitor and H^+^-ionophore could create an auto-enhancing cycle of acidification that would specifically target tumor cells. The specificity of this treatment regime for cancer cells would come from (i) their reverse pH gradient and (ii) that they rely mostly on glycolysis as a source of ATP (the Warburg effect), thereby producing acidifying lactic acid. Upon ionophore-induced extra-to-intracellular proton leakage, cancer cells would consume more glucose to aliment proton pumps counteracting acidification, thus producing even more lactic acid, and thereby escalating cancer cells into hyper-acidification and ATP depletion. We named a principle comprised by this cycle as a ‘Warburg Trap’ (Fig. 4e).

**Fig. 5:**
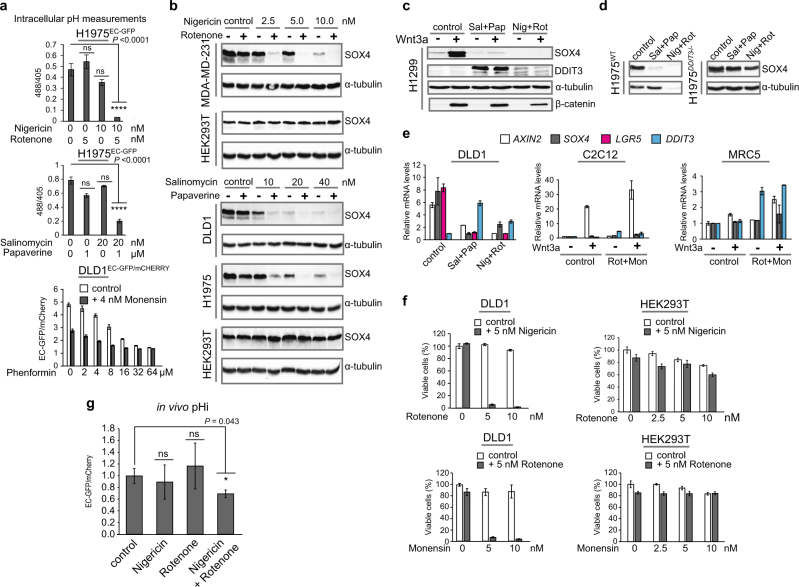
Mitochondrial complex I inhibitors cooperate with ionophores for targeted Wnt signaling repression in tumor cells. **a** Intracellular pH monitored by live imaging of the indicated cell lines treated with the indicated drug combinations, 48 h. **a, e**–**f** Error bars represent mean values ± SD; *P* *≤* 0.0001 (****). **b**–**d** Levels of the indicated proteins were monitored by WB in cell lines treated with drug combinations. **c**–**d** Treatment with 10 nM Salinomycin (Sal), 1 µm Papaverine (Pap), 5 nM Nigericin (Nig), and 5 nM Rotenone (Rot), for 72 h. **e** qRT-PCR analysis of the indicated mRNAs in the indicated cell lines treated with combination of Salinomycin (Sal), Papaverine (Pap), Nigericin (Nig), Rotenone (Rot) (as in **c**–**d**), and 5 nM Monensin (Mon). **f** Cell viability assessed in the indicated cell lines treated with drug combinations, as indicated. **g** In vivo pHi measurements done by live imaging of nude mice xenografted with DLD1^EC-GFP/mCHERRY^ cells (n = 5 mice/group) and treated with Nigericin/Rotenone (*P* *=* 0.043 (*)). Error bars represent median values ± SEM; ns - not significant.

We tested this hypothesis by treatment of cancer and non-cancer cell lines with selected drug combinations: Nigericin (ionophore) with Rotenone (MCI inhibitor), Salinomycin (ionophore) with Papaverine (MCI inhibitor), and Monensin (ionophore) with Phenformin (MCI inhibitor). We confirmed that these drug combinations lowered pHi in vitro (Fig. 5a, and Supplementary Fig. S9a). Predictably, the drug combinations cooperatively blocked constitutive and Wnt3a-induced SOX4 expressions in cancer cell lines (Fig. 5b-c, and Supplementary Fig. S9b-c), and this effect was rescued in *DDIT3*^*−/−*^ cells (Fig. 5d).

Additionally, we observed that DDIT3 was expressed only transiently. When cells were treated with the drug combinations for 24 h, DDIT3 protein induction was detected simultaneously with SOX4 repression in H1299 cells. However, 24 h later (at 48 h post-treatment), DDIT3 was undetectable (Supplementary Fig. S9c). Importantly, SOX4 remained repressed, even in the absence of detectable DDIT3.

Then, the drug combination treatment blocked aberrant Wnt signaling in DLD1 cells bearing *APC* mutation, with no effect in non-cancer cell lines (C2C12 and MRC5), manifested by strong decrease of expression of Wnt target genes, *AXIN2* and *LGR5*. *LGR5* is a colon and lung cancer stem cell marker^55,56,^ and its expression reflects stemness-like properties of cancer cells (Fig. 5e and Supplementary Fig. S9d). Moreover, we confirmed that the drug combinations reduced the ATP levels in cancer cells, but not in non-cancer cell lines (HEK293T and MRC5, Supplementary Fig. S9e). DDIT3 is also a potent inducer of apoptosis^48^, and the selected drug pairs strongly cooperated in reducing viability of various cancer cell lines of different origin, with no substantial effect in non-cancer cell lines (Fig. 5f, and Supplementary Fig. S10a). We ruled out that this effect was mediated by reactive oxygen species (ROS) (Supplementary Fig. S10b).

**Fig. 6:**
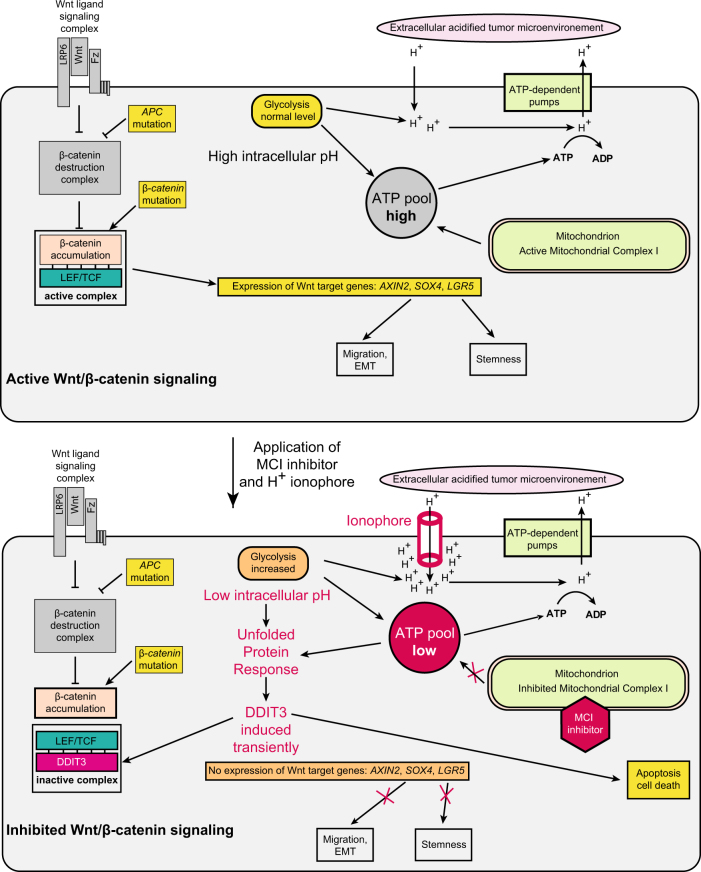
A mechanism of Wnt/β-catenin signaling repression by treatment with MCI inhibitor and H^+^ ionophore combination. Without treatment (upper cell), Wnt/β-catenin signaling can be triggered either with Wnt ligand, or by inactivating *APC* mutation, or with stabilizing *β-catenin* mutation, all leading to β-catenin accumulation. β-catenin binds LEF/TCF transcription factors and induces target genes. Intracellular input of acid produced by weak leakage of protons through the cell membrane and due to glycolysis is compensated by the ATP-dependent H^+^ pumps. The proton pumps are fueled with the ATP produced by glycolysis and the TCA cycle in mitochondria. Once drug combination is applied (lower cell), upon a treatment with ionophore, leakage of protons from outside increases. Simultaneously, ATP production by the TCA cycle in mitochondria is reduced due to MCI inhibition. Compensatory increase of glycolysis produces even more acid causing acidification of intracellular compartment and thus, more ATP consumption that leads to ATP deficit. The resulting condition induces ER stress and the UPR, including transient DDIT3 induction. DDIT3 prevents β-catenin/LEF/TCF complex formation and its interaction with promoters of target genes leading to blockage of Wnt/β-catenin signaling, despite of β-catenin accumulation. If intracellular acidification and ATP depletion persist, DDIT3 induces apoptosis and death of cancer cells

Finally, the Nigericin/Rotenone combination lowered pHi in vivo *(P* *=* 0.043*)* in a xenograft tumor model with DLD1^EC-GFP/mCHERRY^ colon cancer cells (Fig. 5g), therefore, providing an additional evidence that the drug combination utilized the same mechanism in vivo that was found for Metformin (Fig. 4c). Mechanistic interactions of the components causing inhibition of Wnt signaling are summarized in Fig. 6. Combination of dual effects on cellular membrane property and energy metabolism provides a unique opportunity for cancer cell specific targeting. Upon treatment (that lasts about 3 days for the most of the tested cell lines), differences between normal and cancer cells become more and more pronounced in terms of ATP level and pHi. We assume that this causes multiple proteins misfolding in the acidified intracellular compartment inducing the UPR and DDIT3, blocking Wnt signaling and activating apoptosis.

## Discussion

In this study, we established that SOX4 is induced by Wnt signaling in number of human cancer cell lines of different entities (Fig. 1). It has been shown before that SOX4 also mediates TGFβ signaling during cancer progression^57,58^. This relevance to cancer development, involvement in at least two major cancer cell signaling pathways ˗ Wnt and TGFβ ˗ makes SOX4 a good candidate to be used as a marker gene suitable for assessment of anticancer treatments. Using SOX4, we discovered that Metformin acts as Wnt signaling inhibitor (Fig. 2 and Fig. 3). We also revealed an ability of Metformin to trigger pHi drop in live cells in vitro and in vivo (Fig. 4a-c). We speculate that this effect underlies the universal anti-cancer property of Metformin. Despite earlier observations concerning acidification caused by Metformin^49,59,^ its ability to induce DDIT3^44^, its MCI inhibitor property^60,^ and ability to deplete ATP^15^, a thorough analysis of intracellular pH dynamics upon Metformin treatment had not been performed. Applying live cell intracellular pH sensor assay, we found that any other tested MCI inhibitors also induced pHi drop (Supplementary Fig. S7a). A straightforward conclusion about reduction of ATP levels due to inhibition of MCI^15^ led us to an idea that ATP depletion would therefore impair defendant proton pumps function. This in turn would lead to misbalance between H^+^ leakage from outer acidified microenvironment to intracellular compartment and enhanced requirement for extrusion H^+^ to outer space. We can speculate that a similar effect on pHi, albeit to lesser extent, could also underlie the anti-diabetes properties of Metformin. Consistent with this idea, it has been reported that other MCI inhibitors also have anti-diabetic properties^61^.

Additionally, we found that inhibition of Wnt signaling by intracellular acidification was not exclusive to MCI inhibitors. Inhibition of lactic acid extrusion by a broad MCT inhibitor was able to lower pHi and block Wnt signaling (Supplementary Fig. S8). This suggests that any treatment resulting in the pHi drop would also block Wnt signaling.

Mechanistically, intracellular acidification inhibits Wnt signaling *via* the Unfolded Protein Response linked to the drop of ATP level. Intracellular acidification induces DDIT3, an endogenous Wnt signaling inhibitor (Fig. 3 and Supplementary Fig. S4a-c), which disrupts the TCF4/β-catenin activation complex (Fig. 2d). In addition, upon acidification, binding of TCF4, as well as CBP/p300, to *SOX4* promoter was impaired (Fig. 2). These events blocked the Wnt response, and importantly, regardless of β-catenin accumulation (Fig. 1a and Fig. 5c), possibly involving β-catenin-independent Wnt/STOP signaling^62^. DDIT3 induction upon drug treatments was transient (Supplementary Fig. S9c), while the inhibition of Wnt signaling was persistent. This suggests that additional components of the UPR or the apoptotic cascade induced by DDIT3^63^ are involved in persistent Wnt signaling suppression and growth inhibition in cancer cell lines (Fig. 5f and Supplementary Fig. S10a).

Previously, Metformin’s suppressive effect on triple negative breast cancer cells was linked to KLF5 transcription factor degradation through drop of cAMP and inactivation of PKA^64^. Intracellular acidification and ATP depletion could be upstream events that target to degradation not only SOX4 but also other transcription factors, such as KLF5.

Based on insights into a link between MCI inhibitors and intracellular pH, we suggested an auto-enhancing cycle inflicted by a combination of ionophore and MCI inhibitor drugs, which would specifically entrap cancer cell,  - a ‘Warburg Trap’. Why would MCI inhibitors affect glycolysis-addicted cancer cells? The phenomenon  of cancer cells being more sensitive to mitochondrial inhibitors compared to non-cancer ones does not have a clear explanation yet^65^. Recently, it has been found that in cancer cells the ratio between glycolysis and OXPHOS rates depend on pHe: the more acidic pHe is in tumor environment, the further cellular metabolism is shifted towards OXPHOS from glycolysis^66^. Similarly, it has been observed that local lactic acidosis in tumor tissue promotes a transition to a non-glycolytic mode^67^. MCI inhibitory component of the Warburg Trap restricts cancer cell from escaping towards OXPHOS, when the microenvironment is acidified. Upon such a treatment, tumor cells experience both a low pHi and drop in ATP level, leading to cell death.

We provide a molecular mechanism for the Warburg Trap action: the pHi drop induced by ionophore drug is accompanied by loss of ATP (Supplementary Fig. S9e). This in turn induces ER stress with subsequent DDIT3 induction by the UPR (Fig. 5c). The role of DDIT3 as endogenous Wnt inhibitor is currently underestimated because of misinterpreted observation on the absence of clear loss-of-function (LOF) phenotype in development of *Xenopus laevis*^45^ and mouse embryos^68^. Indeed, *DDIT3* LOF was found to have an effect only upon ER stress. Consistent with this, DDIT3 mediates Metformin and the Warburg Trap effects only once the UPR is activated (Fig. 3a-c and Fig. 5d).

While our results strongly support a conclusion about specificity of the Warburg Trap towards cancer cells, the results suggest that this was not exclusively *via* Wnt inhibition, since *SOX4* was repressed regardless of whether its expression is controlled by Wnt or other cell signaling pathways, such as TGFβ (Fig. 1b-c and Supplementary Fig. S1e), and this is consistent with DDIT3 being a global transcriptional repressor with a range of effects. Yet, Wnt signaling might be more sensitive to the treatment by Metformin and the Warburg Trap drugs in comparison to other pathways, since it is predominantly affected in diabetic lung cancer patients taking Metformin (Fig. 1f-g and Supplementary Fig. S2), and those with Wnt-addicted tumors had prolonged survival upon Metformin intake (Fig. 1g). We therefore suggest that post-surgery treatment with MCI inhibitors alone or as the Warburg trap drug combinations could be considered as a supportive therapy component, especially in cases of patients with tumors scored high for Wnt signaling, to prevent their tumor recurrence risk. This would be in agreement with earlier observations on beneficial effect of Metformin treatment for cancer patients^69^. A selective action of Metformin on Wnt-dependent tumors may help explain discrepancy in studies demonstrated reporting on no or negative effects of Metformin intake in diabetic cancer patients, e.g., in case of non-small cell lung cancer, NSCLC^9^.

The main limitation for use of Metformin as an anticancer drug is a requirement to apply it at very high concentrations to achieve a significant impact in cancer cells in vitro. As an alternative MCI inhibitor, Phenformin might also be considered. It was found to be well tolerated, and in our experiments was effective at lower concentrations than Metformin (Fig. 5a, and Supplementary Fig. S10a).

Could H^+^ ionophores have a future to be used for cancer patients treatment? The H^+^ ionophore Monensin is approved by FDA for application in veterinary as an anticoccidosis drug, and it displays no toxic effects in many different animals, with concentrations reaching 220 nM in plasma^70^ (that is 40 times higher than the concentrations applied in experiments described here (Fig. 5f, and Supplementary Fig. S10a)). Another anticoccidosis drug, H^+^ ionophore Salinomycin, has been already tested on cancer patients, and encouraging results have been reported^71^. Collectively, these data suggest that H^+^ ionophores have a good potential to be applied in human. Of note, application of H^+^ ionophores, Nigericin and Salinomycin, has been already suggested for cancer stem cells targeting, and these drugs have been demonstrated to sensitize cancer stem cells^72^. Also, Salinomycin treatment has shown some promising results in medical practice^71^. However, a specificity of such a single treatments remained unresolved.

Our provision of a molecular mechanism for the Warburg Trap raises the possibility to develop multiple further applications, which could also be combined with routine chemo- and radio-therapies. We speculate that the Warburg Trap could be used to overcome multiple drug resistance caused by ATP-dependent extrusion pumps. Such pumps (ABC transporters^73^) cannot be easily inhibited since they are required for normal cells de-toxication. However, the Warburg Trap approach could overcome this obstacle and provide targeted inhibition. Of note, ABCB1 and ABCG2 are Wnt-inducible transporters^74^ and therefore might be even more sensitive to the Warburg Trap inhibition.

In addition, the Warburg Trap principle for targeted anticancer drug design provides a basis for developing new-formula drugs with optimal pharmacological properties. Our findings therefore set a solid basis for in vivo experiments and development of pre-clinical investigations that would follow this study.

Collectively, our data provide the mechanistic rationale for a combination drug-based cancer cell specific approach to inhibit Wnt signaling downstream of β-catenin. This approach offers a new strategy for cancer cell specific treatment, especially for Wnt-addicted tumors such as a colon and lung cancer.

## Materials and methods

### Cells, constructs, chemicals, general procedures

Human cell lines of lung: H1299, H1975, H1703, A549; colon: HCT116, DLD1; breast MDA-MB-231, and prostate PC3 cancers, glioblastoma-like U87MG and melanoma A375 were grown in RPMI (Lonza), supplemented with 11 mg/ml sodium pyruvate (Lonza). Murine B16F10 (melanoma) and C2C12 (immortalized normal myoblast) cell lines, immortalized non-cancer human fetal lung fibroblast-like MRC5, non-cancer human embryonic kidney epithelial HEK293T, HEK293 and Phoenix Ampho human cell lines were grown in DMEM (Lonza). Culture media for all cell lines were supplied with 10% fetal calf serum (FCS; Biosera), and 11 mg/ml penicillin and streptomycin (PAA Laboratories GmbH). For cancer cell lines, to imitate the pHe of tumor microenvironment, cell culture media were additionally supplemented with 20 mM PIPES and adjusted to pH 6.5; for non-cancer cell lines, to imitate pHe of non-tumor microenvironment, cell culture media were additionally supplemented with 20 mM HEPES and adjusted to pH 7.5.

Expression vectors for human *LRP6*, *Xenopus tropicalis β-catenin*, TOPFLASH-luciferase reporter were described before^75^. Xenopus-*wnt8*-human-*frizzled5* fusion construct^76^, human *DDIT3*-luciferase reporter containing two copies of the C/EBP-ATF binding site in the AARE (*DDIT3*-Luc)^77^ and expression vector for Ecliptic *EC-GFP*^51^ were described before. p*Renilla*-TK (Promega) was a commercial construct. Human *DDIT3* expression vector was generated by cloning *DDIT3* ORF obtained by RT-PCR using primers listed in Supplementary Table S2 from H1299 cells grown in the presence of Metformin for 48 h, in pCS2^+^ vector at ClaI/XbaI restriction sites. It also was sub-cloned into a retroviral expression vector with a Tet-inducible promoter pMOWSIN-TREl^78^. Resulting construct, pMOWSIN-TREl-*DDIT3*, was used in combination with pMOWS-rtTAM2 encoding the cDNA for trans-activator protein for retroviral transduction of selected cell lines using Phoenix Ampho packaging cell line, as described before^79^, and individual positive clones were selected with Puromycin, 48 h after transduction. To induce DDIT3 production, cells were treated with 5 µg/ml Doxycycline for 72 h. To create cells expressing pH-sensitive GFP variant, *EC-GFP* was sub-cloned in pMOWSneo-MCS retroviral vector^80^ at BamHI/EcoRI sites (with primers listed at Supplementary Table S2). Transduced cells (H1975 and HEK293) were selected with Neomycin, 48 h after transduction. To create pMOWS-*EC-GFP/mCherry*, Neo-resistance cassette was removed from pMOWS-Neo-*EC-GFP* construct with AfeI/HindIII and substituted with HindIII/ApaI-blunt fragment of *mCherry* from pcDNA3.1-*H2b*-*mCherry* (a gift from Robert Benezra (Addgene plasmid 20972)). The resulting construct was used for retroviral transduction in DLD1, and individual positive clones were screened for co-expression of GFP and mCherry with fluorescence microscope.

Induction with Wnt3a was performed by adding conditioned media (WntCM) at 1:4 ratios to the culture media for 48 h or as indicated, as described before^75^.

### Drug treatments in cell culture

In all experiments, unless stated otherwise, treatments were done using following conditions. Mitochondrial complex I inhibitors: Metformin - 6 mM for 72 h; Rotenone - 5 nM for 48 h; Papaverine HCl - 0.5 µM for 72 h or 1 µM for 48 h; Phenformin - as indicated, for 72 h; Bay 87-2243 - as indicated, for 48 h. Ionophores: Nigericin - 10 nM for 48 h; Salinomycin - 10 nM for 72 h; Monensin - 5 nM for 48 h. Other drugs were used at following concentrations and time periods, unless stated otherwise: Bortezomib - 20 nM for 48 h; Tunicamycin - 1–5 µg/ml for 48 h. Graviola leaves extract was prepared, as described before^81^ (100 mg/ml concentration corresponds to 100 mg leaves extracted with 900 mg DMSO) from powdered dry leaves of plant *Annona muricata* (Moringa Shop, Germany), applied at concentrations 0.01–0.16 µg/ml, for 72 h.

### 3D collagen invasion assay

3D collagen gels were prepared as described^82^. In brief, ice-cold 1 M HEPES buffer, 0.7 M NaOH, 10× PBS pH 8.0 and bovine skin collagen G solution (L1613, Biochrome) were mixed in 1:1:2:16 ratios, respectively. 35 μl of the resulting solution were added per well of a flat bottom 96-well plate (BD 353376). A plate was kept overnight at 4 °C to allow gelation of the collagen. After gelation, 10,000 cells per well were seeded on top of the matrix, cultured overnight and stimulated with WntCM. 96 h later, cells were fixed with 3.7% PFA for 1 h and stained with Hoechst (Sigma). Imaging was performed using a LSM710 confocal microscope (Carl Zeiss) equipped with EC Plan-Neofluar DIC 10×/0.3 NA objective lens (Carl Zeiss). For each well, a 2 × 2 tile z-stack was acquired. Image analysis was performed using Imaris software (Bitplane). Spots detection algorithm was applied to assign a spot for fluorescent intensity of each individual nucleus. Resulting spots were filtered by their z-position to separate collagen invaded cells from the cells remained on top of the matrix. Percentage of invaded cells was used as an output.

### Optical measurements of intracellular pH

The assay was done as described^83^. In brief, cell lines expressing pH-sensitive EC-GFP or EC-GFP/mCherry, were densely seeded on 96-well flat-bottom plates (BD353376). The next day, cells were treated with drugs for 48 h, or as indicated. Live cells were imaged using LSM710 confocal microscope (Carl Zeiss) equipped with EC Plan-Neofluar DIC 10×/0.3 NA objective lens (Carl Zeiss). For each well, 2 × 2 tile was acquired. In case of H1975^EC-GFP^ and HEK293^EC-GFP^ cell lines, 405 nm and 488 nm lasers were used for EC-GFP excitation, and emission light was collected using 535/50 filter for each laser. 488/405 ratios were quantified using ImageJ (NIH) software. In case of DLD1^EC-GFP/mCHERRY^ cell line, 485 nm (EC-GFP) and 538 nm (mCherry) excitation filters and 510 nm (EC-GFP) and 620 nm (mCherry) emission filters were used to collect EC-GFP and mCherry signals, respectively, using Fluoroscan Ascent microplate reader (Thermo Fisher Scientific). To convert 488/405 and 485/538 (EC-GFP/mCherry) ratios to pHi values, a calibration curve for each cell line was produced, as described^84^. In brief, untreated cells were exposed for 30 min to high K^+^ solutions with different pH (6.2–8.8 range) (140 mM KCl, 1 mM MgCl_2_, 2 mM CaCl_2_, 5 mM glucose buffered with either 40 mM MES or 40 mM Tris) containing 5 µM Nigericin (Supplementary Fig. S7b, and data not shown). Each pHi measurement experiment was done with 5–7 biological replicates for every treatment.

### PLGA microspheres formulations preparation

To ensure a continuous drug release and avoid high peak doses after bolus injections into animals, we used biodegradable polymer, PLGA (poly (lactic-co-glycolic acid)), as vehicle for the drug formulations. PLGA (lactid: glycolide (75:25)) (SIGMA or Vornia Biomaterials) formulations consisted of drug in the range of 5–20 mg per 1 g of PLGA, as described elsewhere^85,86^. In brief, 100 mg of PLGA beads were dissolved in 5 ml Dichloromethane (DCM) together with the selected drug. PLGA/drug mixture was added drop-wise to 15 ml of ice-cold 0.4% PVA (Polyvinylalcohol) water solution on ice, mixed for 1 h by rotation (600 rpm) using magnet mixer. Formed emulsion was then left at room temperature on a mixer until DCM was evaporated (overnight) and subsequently washed three times with distilled water. Obtained microsphere beads were segregated according to their size by centrifugation for 5 min at 209 *g*, followed by separation using a 250 µm cell strainer (Thermo Fisher Scientific). After pelleting, beads were air-dried and additionally stored in vacuum for at least 24 h, to ensure removal of traces of DCM. Finally, the beads were weighted and resuspended in sterile PBS for injections into animals.

### Animal experiments

Animal care and all animal experiments were performed according to the national guidelines and were approved first by an institutional review board/ethics committee headed by the local animal welfare officer (Dr. Michaela Socher) of the German Cancer Research Center, Heidelberg, Germany. All experiments were in addition approved by the responsible national authority, the local Governmental Committee for Animal Experimentation (Regierungspräsidium Karlsruhe, Germany) under licenses G244/11, G284/15, G195/16, and were carried out accordingly.

### In vivo and ex vivo pHi measurements in xenograft tumors

Five to six-week-old female NMRI-Foxn1^nu^ (Charles River, Sulzfeld, Germany) were subcutaneously injected with 2 × 10^6^ H1975^EC-GFP^ human lung cancer cells, resuspended in 100 µl PBS into the right flank. Eighteen days after transplantation, once the tumor growth was detected, mice received drinking water containing 200 µg/ml Metformin or normal drinking water, as a control, for 7 days. In vivo and ex vivo tumor imaging was performed with IVIS Lumina III (Perkin Elmer) system using 460 nm excitation and 520 nm emission filters and quantified with LivingImage software V4.4 (Caliper Life Sciences). For the experiment, in which pHi changes were induced with a combination of an ionophore and a MCI inhibitor, nude female mice were subcutaneously injected with 2 × 10^6^ DLD1^EC-GFP/mCHERRY^ cells resuspended in 100 µl PBS. When the tumor diameter reached 5 mm, mice were in vivo imaged with IVIS Lumina III, and the initial control GFP/mCherry ratios in  xenografted tumors were measured using the appropriate filter sets. Animals were randomized into four cohorts (n = 5 mice in each group), and were intraperitoneally injected, twice per week, either with 10 mg of control PLGA microsphere preparations in 200 µl PBS alone, or with 5 mg PLGA containing either 5 µg Nigericin/mg PLGA or 20 µg Rotenone/mg PLGA beads, in 100 µl PBS, or the combination of both compounds (10 mg of beads in 200 µl PBS). One week later, animals were subjected to in vivo imaging, as described above. During live imaging, the animals were anesthetized with 5% initiating and 2% maintaining doses of isoflurane. EC-GFP/mCherry values were normalized to the control treatment and initial control signals.

### qRT-PCR

RNA and cDNA were prepared as described^87^, and qRT-PCR was performed with UPL probes (Roche) using LC480 LightCycler (Roche). Details of qPCR primers and UPL probes used for amplification are provided in Supplementary Table S2. mRNA values were normalized to the housekeeping gene *GAPDH* (all in vitro experiments), or *ESD* (patients’ samples).

### Western blot analysis (WB)

Cells were lysed in Triton lysis buffer (TBS (50 mM Tris pH7.4, 150 mM NaCl, 2.7 mM KCl), 1% Triton X-100, 2 mM β-mercaptoethanol (ME), 1 mM MgCl_2_, 1 × proteinase inhibitor cocktail (PIC) (Sigma)) for 5 min on ice. Lysates were cleared by centrifugation and analyzed by SDS-PAGE. For β-catenin analysis, cytosolic extracts were prepared using Saponin lysis buffer (0.05% saponin, 1 mM MgCl_2_, 1 × TBS, 2 mM ME, 1 × PIC), for 30 min on ice. Antibodies used were: SOX4 (Diagenode cs-129-100), α-tubulin (Thermo Scientific, MS-581-P0), DDIT3 (Santa Cruz sc-575), β-catenin (BD Transduction Laboratories, 610153), and TCF4 (Santa Cruz, sc-166699).

### ChIP assay

Chromatin immunoprecipitation assays were done essentially as described^88,^ with some modifications. In particular, after fixation of cells with 1% formaldehyde for 10 min, nuclei preparation and washing, chromatin was sheared in Shearing buffer (25 mM Tris-HCl, pH 8.0; 1 mM EDTA, 0.5 mM EGTA, 2.5 mM sodium pyrophosphate, 1 mM PMSF, 1 × PIC) using Covaris S2 (ultrasonicator with following settings: Time = 20 min, 20% Duty Cycle, Intensity = 4, 200 cycles per burst), or with Sonoplus Bandelin (12 times for 30 s, 40% power). Before immunoprecipitation, Triton X-100 to 1%, SDS to 0.1% and NaCl to 150 mM were added to the sheared chromatin. IP reactions were incubated overnight, and formed immunocomplexes were recovered using protein A/G magnetic beads (Pierce). After washings, complexes were eluted with Proteinase K digestion buffer (20 mM HEPES, pH 8.0, 200 mM NaCl, 1 mM EDTA, 0.5% SDS), and immunoprecipitated DNA was recovered after digestion with 400 µg/ml Proteinase K for 1 h at 42 °C, RNase A treatment and crosslinking reversal. DNA was purified and analyzed by qPCR analysis using SYBR Green master mix (Qiagen QuantiTect) with primers listed in Supplementary Table S2. CBP and p300 antibodies were from Santa-Cruz Biotechnology (sc-369X  and sc-585X , respectively), TCF4 and normal IgG were from EMD Millipore (17-10109 and 12-370, respectively).

### Co-immunoprecipitation assay (Co-IP)

Cells were lysed in Triton lysis buffer (TBS (50 mM Tris pH7.4, 150 mM NaCl, 2.7 mM KCl), 1% Triton X-100, 1 mM MgCl_2_, 1 × proteinase inhibitor cocktail (PIC) (Sigma)) for 20 min on ice. Lysates were cleared by centrifugation at 2000 g and subjected to immunoprecipitation with TCF4 antibody (Millipore, 17-10109) bound to Protein A/G-PLUS agarose beads (Santa Cruz, sc-2003) overnight. After washing beads 6 times with washing buffer (TBS, 0.1% Triton X-100, PIC), immunocomplexes were eluted with Laemmli Sample Buffer 4× (Bio-Rad), resolved in SDS-PAGE and analyzed by WB.

### Patients' tumor samples

All patients underwent a surgery at the Thoraxklinik, University Hospital Heidelberg.

RNA samples from lung tissues were provided by LungBiobank Heidelberg, a member of the BioMaterialBank Heidelberg (BMBH), and the biobank platform of the German Center for Lung Research (DZL). All patients gave written informed consent for the use of their biomaterials for research purposes. The protocol was approved by the ethics committee of the University of Heidelberg. Tissues were snap-frozen within 30 min after resection and stored at −80 °C until the time of analysis. Tumor histology was classified according to the 3rd edition of the World Health Organization classification system^89^. A brief summary of patients’ clinical data is presented in Supplementary Table S1.

### Isolation of tissue samples RNA

Total RNA was isolated using an RNeasy Kit (Qiagen, Hilden, Germany) according to the manufacturer’s instruction, with the DNase I treatment step included. The quality of total RNA from patients’ tissues was assessed with an Agilent 2100 Bioanalyzer and Agilent RNA 6000 Nano Kit (Agilent Technologies, Boeblingen, Germany). RNA was considered sufficient for further analyses if it had an RNA integrity number (RIN) of at least 8.0.

### Luciferase reporter assays

Luciferase reporter assays were carried out in 96-well plates, as described before^75^. In brief, a total of 100 ng of DNA was transfected per well, including either 5 ng TOPFLASH with 1 ng p*Renilla*-TK or 10 ng human *DDIT3*-luciferase reporter (*DDIT3*-luc) and, when indicated, 4 ng human *LRP6*, 4 ng Xenopus-*wnt8*-human-*frizzled5* fusion, 0.5 ng *Xenopus tropicalis β-catenin* and 70 ng human *DDIT3*. Drug treatments were applied next day after transfection, and cells were treated for 48 h prior to luciferase activity measurements with the Dual luciferase system (Promega).

### Cell viability assay

Indicated cells were treated with indicated drug combinations for 48 h in conditions imitating tumor environment, in case of cancer cell lines, or in conditions of non-tumor environment, in case of non-cancer cell lines. After the treatment, cells were analyzed for cell viability using CellTiter-Glo® Cell Viability Assay (Promega), according to the manufacturer’s recommendations. For each treatment, fractions (%) of viable cells were quantified, with untreated viable cells set as 100%. Each experiment was repeated at least twice, with 6 biological replicates for each condition.

### Measurement of cellular ATP levels

ATP levels were quantitated by the CellTiter-Glo® Cell Viability Assay kit (Promega), according to the manufacturer’s recommendations. In brief, serial dilutions of ATP solutions were prepared, and the luminescence signal was recorded to build a calibration curve. Cells were treated, as described above for the cell viability assay, and an absolute ATP amount per cell was quantified using the calibration curve. Each measurement was repeated at least twice, with 6 biological replicates for each condition.

### siRNA transfection experiments

For mRNA knock-down experiments, cells in 6-well format were transfected with 50 nM of non-targeting control siRNA or with corresponding ON-TARGET SMARTpool siRNA or siGENOME SMARTpool siRNA (all from Dharmacon, except for *DDIT3* siRNA, which was from Santa-Cruz) using DharmaFECT1 reagent (Dharmacon) following the manufacturer protocol. 24 h after siRNA transfection, the cells were treated with WntCM, and subjected to selected drug treatment, as indicated. Cells were harvested in 72–120 h post-transfection, depending on experiment, for Western blot and qRT-PCR analyses. For each experiment with siRNA transfection, mRNA knockdown efficiencies were quantified using qRT-PCR, and a targeted mRNA levels were found to be reduced down to at least 15–20% of the one in a control treatment with non-targeting siRNA (Supplementary Fig. S4, and data not shown).

### CRISPR-mediated knock-out of DDIT3 gene

H1975 cells were stably transfected with Cas9 under Blasticidin selection (pHCSVBlast-*Cas9*, Dharmacon). Positive clone was selected using detection of Cas9 in WB using Cas9 antibody (Novus NBP2-36440, clone 7A9-3A3). Cas9 expressing cells were transfected with a 1:1 molar ratio mix of tracrRNA (Dharmacon product U-002000-120) and *DDIT3*-specific crRNA (5´CUGGUAUGAGGACCUGCAAGGUUUUAGAGCUAUGCUGUUUUG 3´, designed with CRISPR RNA Configurator tool, Dharmacon) using DarmaFECT1 (Dharmacon). 5 days later, cells were harvested and plated at low density. Individual clones were treated with DDIT3-inducing drugs and assayed by WB, 48 h later (Supplementary Fig. S5e). Clone that failed to produce DDIT3 protein, was selected and verified with successive sequencing. The effective mutation is highlighted in Supplementary Fig. S5e.

### Statistical analysis

GraphPad Prism software was used for most statistical analyses. Two-way ANOVA tests were used to assess statistical significance in cell invasion, pHi properties between treated and untreated cell lines in vitro. In vivo changes of pHi in tumor xenograft tissues were tested with unpaired *t*-test. Cell invasion properties and ATP amount changes were assessed using one-way ANOVA Dunnett’s multiple comparison tests.

Correlations between expression of mRNA of interest and of Wnt target genes in patients’ tumor samples were carried out using non-parametric correlation tests, and Spearman’s rank correlation coefficients were calculated. For assessment of patients’ survival data, Kaplan–Meier curves were built, and log-rank (Mantel–Cox) test was calculated using IBM SPSS statistics software (V22.0). For finding an optimal cut-off level and survival data stratification, ADAM statistical software package (DKFZ, Heidelberg) utilizing Critlevel procedure was used^90^. In brief, for the combined patients cohort (including both Metformin-taking and not taking Metformin lung cancer patients), the optimal expression cut-off levels for the tested genes were calculated and used as new variants (0 = below threshold, 1 = above threshold), and these parameters were applied to assess the survival data. Only for the Metformin-taking lung cancer patients cohort stratification by *AXIN2* expression (with a cut-off at 1.02), and not for the other tested genes, revealed differences in their survival that were found to be significant (*P* = 0.046).

## Electronic supplementary material

Supplementary Information
