# Supplementary material for: Cancer cell specific inhibition of Wnt/β-catenin signaling by forced intracellular acidification
Source: Cell Discov. 2018 Jul 3;4:37. doi: 10.1038/s41421-018-0033-2 (PMC6028397; doi:10.1038/s41421-018-0033-2)
Supplement: Supplementary file 1 — Supplementary Information [file 41421_2018_33_MOESM1_ESM.pdf]

Supplementary Information for:

**Cancer cell specific inhibition of Wnt/β-catenin signaling by forced intracellular acidification**

Svitlana Melnik[1,2,12], Dmytro Dvornikov[3,4,12], Karin Müller-Decker[5], Sofia Depner[3], Peter Stannek[6], Michael Meister[4,7], Arne Warth[4 8], Michael Thomas[4,7], Thomas Muley[4,7], Angela Risch[1, 4,9, 10], Christoph Plass[1], Ursula Klingmüller[3,4], Christof Niehrs[6,11], Andrey Glinka[6*].

*To whom correspondence should be addressed:
E-mail: glinka@dkfz.de

This pdf file includes:

Supplementary Tables 1-2
Supplementary Figures 1-10

**Supplementary Table 1.** Summary of clinical and mRNA expression data for the lung cancer patients cohort used in this study

| **Category** | **Non-diabetic** | **Metformin-taking diabetic** |
|---|---|---|
| Number of patients | 66 | 42 |
| Age, median (range) | 63 (43 – 80) | 69 (53 – 79) |
| Male/Female | 52/14 | 33/9 |
| NS/FS/S/U | 5/35/26/0 | 2/23/16/1 |
| ECOG 0/1/unknown | 55/9/2 | 38/4/0 |
| p-stage I/II/III | 20/24/22 | 9/13/18* |
| **Histology** | | |
| Squamous cell | 26 | 19 |
| Adenocarcinoma | 30 | 16 |
| Large cell | 4 | 2 |
| Other (lung metastases and secondary malignant lung tumors) | 6 | 5 |
| Postoperative chemotherapy | 36 | 16 |
| Postoperative radiotherapy | 18 | 5 |
| Recurrence (local, distant, 2nd tumor) | 29 | 20 |
| Dead | 29 | 15 |
| **mRNA expression,** Mean value ± SEM | | |
| *AXIN2* | 1.89±0.45 | 2.22±0.22 |
| *SOX4* | 3.77±0.45 | 3.01±0.28 |
| *DDIT3* | 4.03±0.58 | 5.09±2.23 |
| *VIM* | 1.59±0.16 | 2.01±0.31 |
| *ZEB1* | 1.30±0.10 | 2.36±0.36 |

* – no stage was assigned
NS – non-smoker
FS – former smoker
S – smoker
U – unknown

**Supplementary Table 2.** List of primers used in this study

| Gene Name | Sequence | UPL | Assay |
|---|---|---|---|
| *DDIT3* | CCCATCGATGCCACCATGGCAGCTGAGTCATTGCC<br>CCCTCTAGATTATGCTTGGTGCAGATTCACC | | Cloning |
| *EC-GFP* | CCCGGATCCGCCACCATGAGTAAAGGAGAAGAAC<br>TTTTCACTGGAG<br>CCCGAATTCTTATTTGTATATTTCATCCATGCCATG<br>TGT | | Cloning |
| *SOX4* | CAACGCCAACTCCAGCTC<br>ACCGACCTTGTCTCCCTTC | 25 | qRT-PCR |
| *AXIN2* | CCACACCCTTCTCCAATCC<br>TGCCAGTTTCTTTGGCTCTT | 36 | qRT-PCR |
| *ESD* | TTAGATGGACAGTTACTCCCTGATAA<br>GGTTGCAATGAAGTAGTAGCTATGA | 27 | qRT-PCR |
| *ZEB1* | TTTTTCCTGAGGCACCTGAA<br>AAAATGCATCTGGTGTTCCAT | 28 | qRT-PCR |
| *GAPDH* | GCATCCTGGGCTACACTGAG<br>AGGTGGAGGAGTGGGTGTC | 82 | qRT-PCR |
| *VIM* | TACAGGAAGCTGCTGGAAG<br>ACCAGAGGGAGTGAATCCAG | 24 | qRT-PCR |
| *DDIT3* | CAGAGCTGGAACCTGAGGAG<br>TGGATCAGTCTGGAAAAGCA | 9 | qRT-PCR |
| *ATF6* | CTTTTAGCCCGGGACTCTTT<br>TCAGCAAAGAGAGCAGAATCC | 42 | qRT-PCR |
| *ATF4* | GGTCAGTCCCTCCAACAACA<br>CTATACCCAACAGGGCATCC | 88 | qRT-PCR |
| *ATF2* | TTTGGTCCAGCACGTAATGA<br>CAAACCCACTTCTTCACAGTTTT | 5 | qRT-PCR |
| *PERK* | CCAGCCTTAGCAAACCAGAG<br>TCTTGGTCCCACTGGAAGAG | 58 | qRT-PCR |
| *IRE1* | TGAGGACGAAGGGGACTACA<br>ACGTCCCCAGATTCACTGTC | 9 | qRT-PCR |
| *GPD2* | CGGACAACGAGAAGTCGTC<br>AGTCCTAAAACAGTTGCAAGAGC | 25 | qRT-PCR |
| *AMPK1* | TCTCAGGAGGAGAGCTATTTGATT<br>GAACAGACGCCGACTTTCTTT | 42 | qRT-PCR |

| | | | |
|---|---|---|---|
| *AMPK2* | ACCCACTGAAACGAGCAACT<br>AGGAAGGGTCTTCAGGAAATAAG | 61 | qRT-PCR |
| *LGR5* | ACCAGACTATGCCTTTGGAAAC<br>TTCCCAGGGAGTGGATTCTAT | 78 | qRT-PCR |
| *DDIT3* | CCCATTATCCTGCAGATGTGC<br>CTTCCTTCAAGGAAATGAGGAAAGG | | genotyping |
| *SOX4 upstream* | ACAGATCCAGTCAGATGGCTAC<br>AGTATGTAGGCTGGCAATAGGC | | qPCR, ChIP |
| *SOX4 promoter (-800)* | TGGGCTATGCAGGATTTACAGG<br>TTGTGCTGTTTGTGGAAGG | | qPCR, ChIP |
| *SOX4 promoter (-400)* | AGCTGGGGAACAGATTTTGC<br>ATAACAAGGG GCTTGGAACG | | qPCR, ChIP |
| *SOX4 exon* | ACATCAAGCGACCCATGAAC<br>TTGTCGCTGTCTTTGAGCAG | | qPCR, ChIP |
| *SOX4 downstream* | TGCCCAATGCCTAAGATTGG<br>AGAGGAAATCTGGCCAAGAGAC | | qPCR, ChIP |
| *GAPDH* | GGGGCCTGTTTTGTTGTCAT<br>GGGCCGTGTGATTCTTTGTT | | qPCR, ChIP |
| *AXIN2*[42] | GGAGCAGTAAAAGGCCGTAA<br>CCAAACCATTGAAGCCCTTA | | qPCR, ChIP |

**Supplementary figure legends**

**Supplementary Figure 1** *SOX4* is a Wnt-inducible gene. (**a-e**) Levels of the indicated mRNA and proteins in the indicated cell lines were monitored by qRT-PCT or WB, respectively. **a,** H1299 cells were treated with: WntCM, epidermal growth factor (EGF, 50 ng/ml), insulin-like growth factor (IGF, 40 ng/ml), basic fibroblast growth factor (bFGF, 1 ng/ml) or insulin (10 μg/ml). Position of unspecific band used as a loading control is indicated by asterisk. Endogenous β-catenin from cytosolic extracts was used as a control of Wnt pathway activation. **b,** H1299 cells were treated with WntCM, as indicated. **c,** Cells were treated with WntCM for 48 h and subjected to siRNA-mediated *β-catenin* knockdown for 76 h. **d,** H1299 cells were transiently transfected with control or *β-catenin*-expressing vectors for 72 h. (**a-d**) Error bars represent mean values ± SD. **e,** Cells were subjected to siRNA-mediated knockdown of *β-catenin* for 48 h.

**Supplementary Figure 2** Metformin affects Wnt signaling in lung cancer patients.

Non-linear Spearman's rank correlation analysis of *VIM* or *ZEB1* mRNA expression levels with Wnt signaling (*AXIN2*) in tumor tissue samples from lung cancer patients, non-diabetic or Metformin-taking diabetic. $\rho$ – Spearman's rank coefficient for non-linear correlation: $P \leq 0.05$ (*); ns – not significant; n – number of patients.

**Supplementary Figure 3** Metformin's effects on SOX4 could not be imitated by interference with GPD2 or AMPK functions. Levels of the indicated mRNAs and proteins were monitored by qRT-PCR or WB, respectively, in the indicated cell lines, subjected to siRNA-mediated knockdown of *GPD2* (**a**) or *AMPK1/2* (**b**) for 96 h, as indicated. Error bars represent mean values ± SD.

**Supplementary Figure 4** DDIT3 is one of the key factors involved in SOX4 repression upon Metformin treatment. (**a-c**) Levels of the indicated mRNA and proteins in the indicated cell lines, treated with WntCM and Metformin, as indicated, were monitored by qRT-PCT or WB, respectively. Asterisk indicates a position of DDIT3 protein band migration. **c,** DLD1 cells were pre-treated with Metformin for 72 h and later transferred into fresh media for the indicated time period. **d,** TOPFLASH luciferase reporter assay in HEK293T cells: Wnt signaling was activated by co-transfection of *LRP6* with *wnt8-frizzled5* fusion constructs (*LRP6/W/Fz*) or by *β-catenin*, in presence of or without *DDIT3* expression construct. Experiment was done in triplicates, error bars correspond to mean values ± SD; RLA – relative luciferase activity. **e,** Confirmation of DDIT3 knockout in H1975 cell line generated with CRISPR/Cas9 genome editing system. Top: Genotyping result showed a single nucleotide insertion mutation (marked by asterisk) in a selected clone that caused translation frame shift. No wildtype alleles were detected for the selected clone after multiple sequencing. Bottom: H1975 wildtype cells and the selected mutant clone were treated with 5 µg/ml Tunicamycin, 60 nM Nigericin or 20 nM Bortezomib for 48 h, and DDIT3 protein induction was monitored by WB.

**Supplementary Figure 5** Efficiency of siRNA-mediated knockdown experiments performed in this study was controlled by qRT-PCR. **a,** Samples from experiment presented in **Fig. 3a**. **d**, Samples from experiment presented in **Fig. 3f**.

**Supplementary Figure 6** The UPR-inducing drugs induce DDIT3 and reduce protein levels of SOX4. (**a-b**) Levels of the indicated proteins in the indicated cell lines treated with WntCM and different drugs were monitored by WB. Asterisk indicates position of unspecific band used as a loading control. **b,** H1299 cells were transfected with *β-catenin* expression construct for 72 h and treated with Metformin or Tunicamycin, as indicated. **c,** 3D cell invasion assay done with H1299 cells treated with WntCM and the indicated drugs (6 mM Metformin or 5 nM Bortezomib) for 96 h. Cells were fixed and imaged using confocal microscopy. Bottom: Representative images of 3D cell invasion assay. Experiment was done with 5 biological replicates, error bars correspond to mean values ± SD.

$P < 0.0001$ (****). Scale bar, 150 µm.

**Supplementary Figure 7** Mitochondrial complex I inhibitors cause intracellular acidification. (**a-c**) Fluorometric measurements of pHi changes (EC-GFP/mCherry) in DLD1$^{EC\text{-}GFP/mCHERRY}$ cells upon treatment with increasing concentrations of the indicated MCI inhibitor drugs for 72 h. **b,** pHi calibration curve built for conversion of EC-GFP/mCherry ratio values to pHi. DLD1$^{EC\text{-}GFP/mCHERRY}$ cells were exposed to standard calibrating solutions with pH range from 6.2 to 8.8, for 0.5 h, before fluorometry. **c,** Treatment was done as in **a**, using increasing concentrations of MCII inhibitor, Lonidamine, in presence or without of 5 nM Monensin. Error bars correspond to mean values ± SD for 6 biological replicates. (**a, c**) Positions of black lines on the right indicate a range of pHi changes caused by the treatments. The pHi values were converted from EC-GFP/mCherry ratios using the calibration curve presented in **b**.

**Supplementary Figure 8** Only when drug treatment causes intracellular acidification, it blocks Wnt signaling. **a,** Fluorometric measurements of pHi changes (EC-GFP/mCherry) in DLD1$^{EC\text{-}GFP/mCHERRY}$ cells upon treatment with increasing concentrations of the indicated monocarboxylate transporter (MCT) inhibitors: CHC (2-Cyano-3-(4-hydroxyphenyl)-2-propenoic acid) and AZD3965, or carbonic anhydrases (CA) inhibitor (Azetazolamide) for 72 h, as indicated. **b,** Levels of indicated proteins were monitored by WB in H1299 cells treated with indicated drugs (as in **a,** for 72 h) and induced with WntCM for 48 h.

**Supplementary Figure 9** Drugs, selected according to the 'Warburg Trap' model, cooperatively drop pHi and inhibit Wnt signaling. **a,** Confocal microscopy in H1975$^{EC\text{-}GFP}$ cells treated with combinations of Rotenone and Nigericin, or Papaverine and Salinomycin, as indicated. Live cells were imaged at 405 nm and 488 nm excitations to record control and pH-sensitive GFP emissions, respectively. Scale bar, 150 µm. (**b-c)** Levels of the indicated proteins in the indicated cell lines treated as in **Fig. 5b** (H1975 and MDA-MB-231, **b**), or as indicated (MRC5, **b**; or H1299, **c**), were evaluated by WB. **d,** qRT-PCR analysis of the indicated mRNAs expression levels in the samples from the experiment presented in **Fig. 5c**. **e,** ATP concentration measurements in the indicated cell lines treated with Rotenone/Nigericin, as indicated. Error bars correspond to mean values ± SD for 6 biological replicates; $P = 0.0042$ (**); ns – not significant.

**Supplementary Figure 10** Drugs, selected according to the 'Warburg Trap' model, cooperatively affect cancer cells viability and inhibit SOX4 protein accumulation, also in presence of ROS scavengers. **a,** Cell viability measured in the indicated cell lines with the indicated drug combinations. Assay was done as in **Fig. 5f**. Error bars correspond to mean values ± SD for 6 biological replicates. **b,** The indicated cell lines were treated with different drug combinations: 10 nM Nigericin (Nig), 5nM

Rotenone (Rot), 1 µM Papaverine-HCl (Pap), 10 nM Salinomycin (Sal), in presence or without of 1mM NAC (N-acetyl cysteine), 20 µM Doxycycline, 20 µM Minocycline, 100µM Trolox (soluble form of vitamin E), as indicated, and levels of SOX4 protein were evaluated with WB.

Melnik *et al.,* Supplementary Figure 1

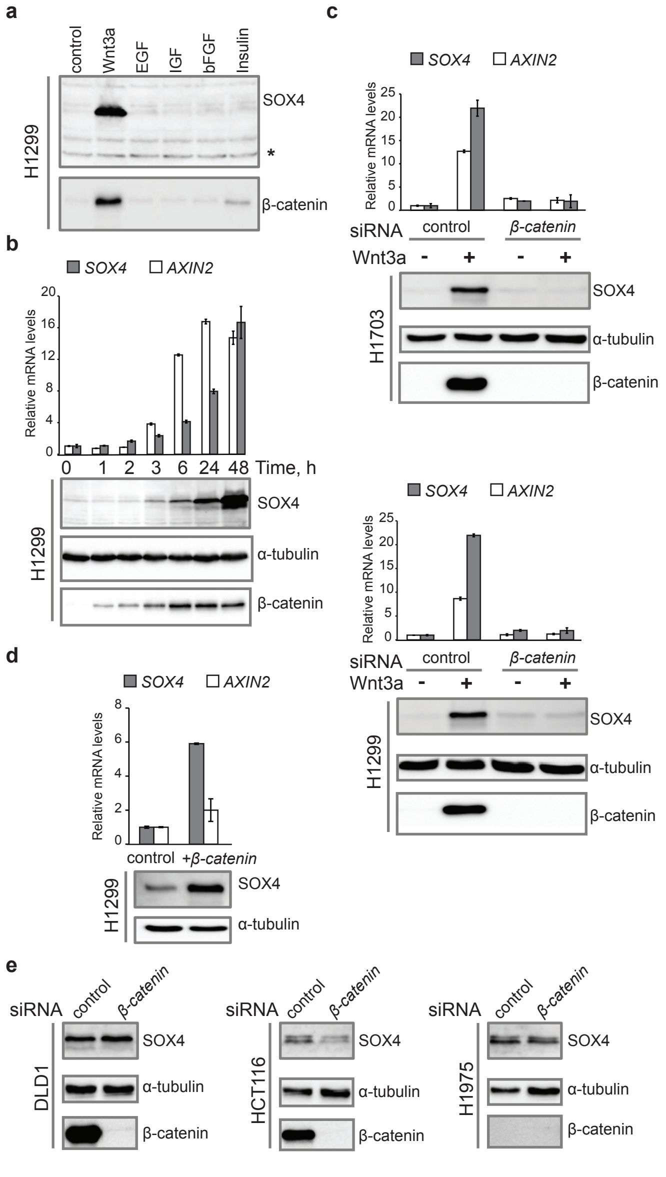

Melnik *et al.,* Supplementary Figure 2

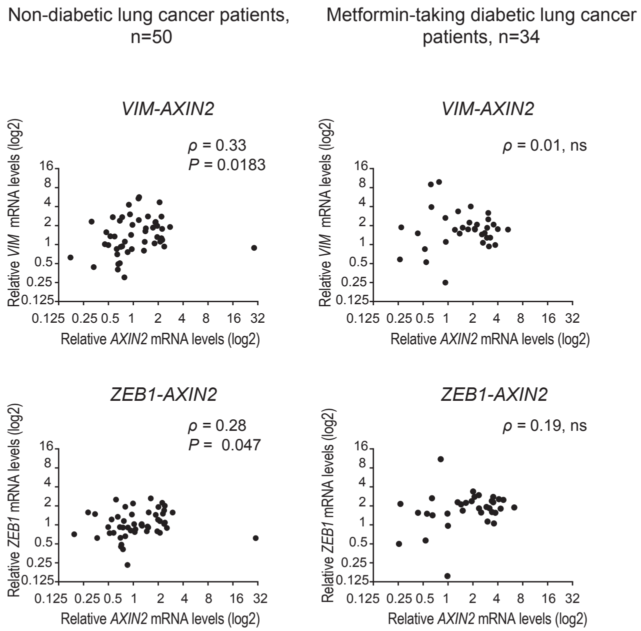

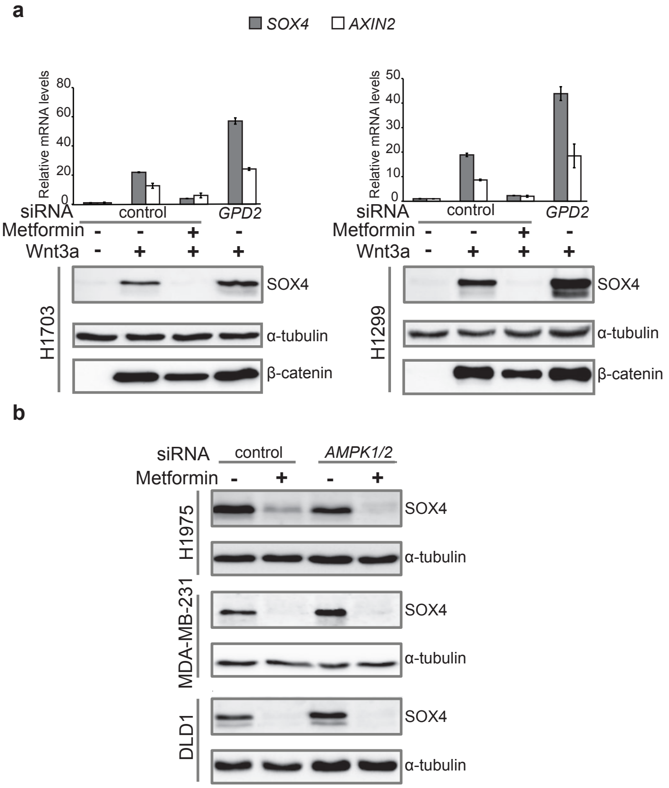
a
SOX4
AXIN2
Relative mRNA levels
80
60
40
20
0
siRNA
control
GPD2
Metformin
Wnt3a
SOX4
α-tubulin
β-catenin
H1703
50
40
30
20
10
0
H1299
b
siRNA
control
AMPK1/2
Metformin
SOX4
α-tubulin
H1975
MDA-MB-231
DLD1

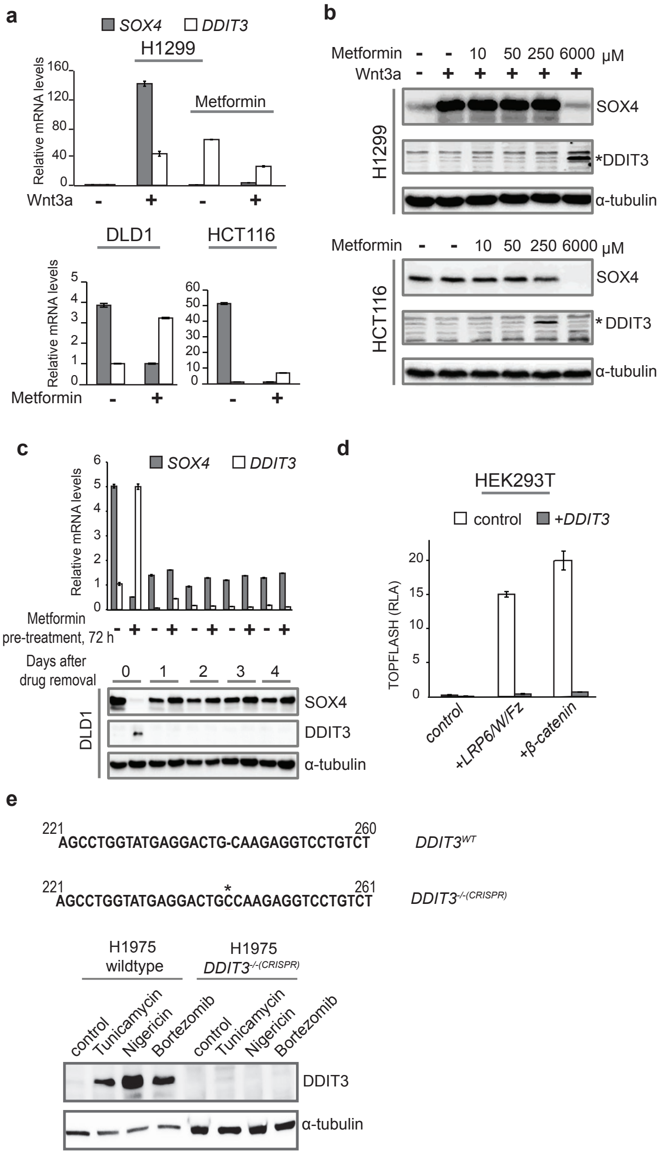
a
SOX4
DDIT3
H1299
Relative mRNA levels
Metformin
Wnt3a
DLD1
HCT116
Metformin
b
Metformin - - 10 50 250 6000 μM
Wnt3a - + + + + +
H1299
SOX4
*DDIT3
α-tubulin
Metformin - - 10 50 250 6000 μM
HCT116
SOX4
* DDIT3
α-tubulin
c
SOX4
DDIT3
Metformin pre-treatment, 72 h
Days after drug removal
0 1 2 3 4
DLD1
SOX4
DDIT3
α-tubulin
d
HEK293T
control
+DDIT3
TOPFLASH (RLA)
control
+LRP6/W/Fz
+β-catenin
e
221 AGCCTGGTATGAGGACTG-CAAGAGGTCCTGTCT 260
DDIT3WT
221 AGCCTGGTATGAGGACTGCCAAGAGGTCCTGTCT 261
DDIT3-/-(CRISPR)
H1975 wildtype
H1975 DDIT3-/-(CRISPR)
control
Tunicamycin
Nigericin
Bortezomib
DDIT3
α-tubulin

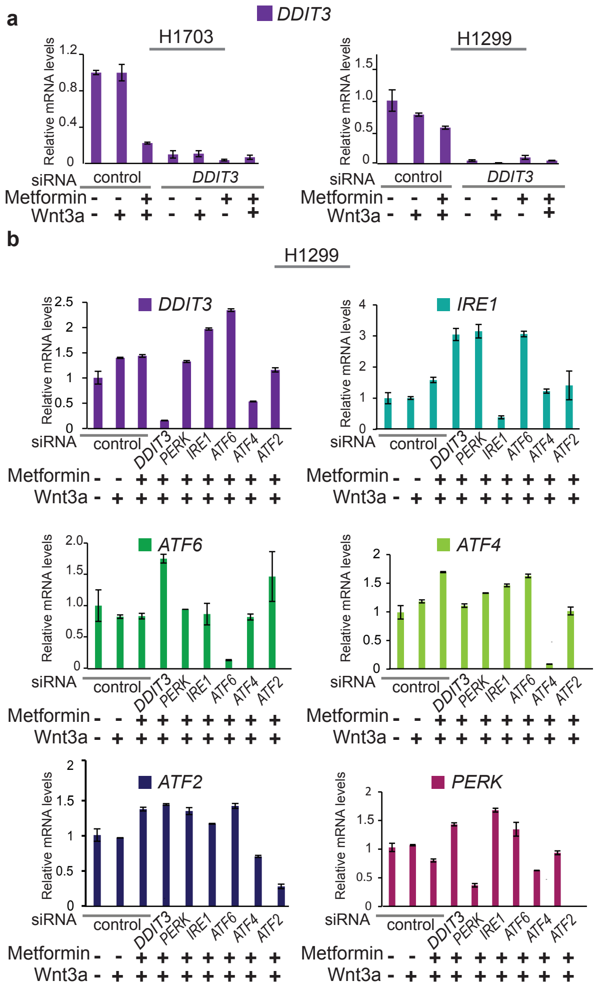
a
DDIT3
H1703
H1299
Relative mRNA levels
siRNA
control
DDIT3
Metformin
Wnt3a
b
H1299
DDIT3
IRE1
ATF6
ATF4
ATF2
PERK
control
PERK

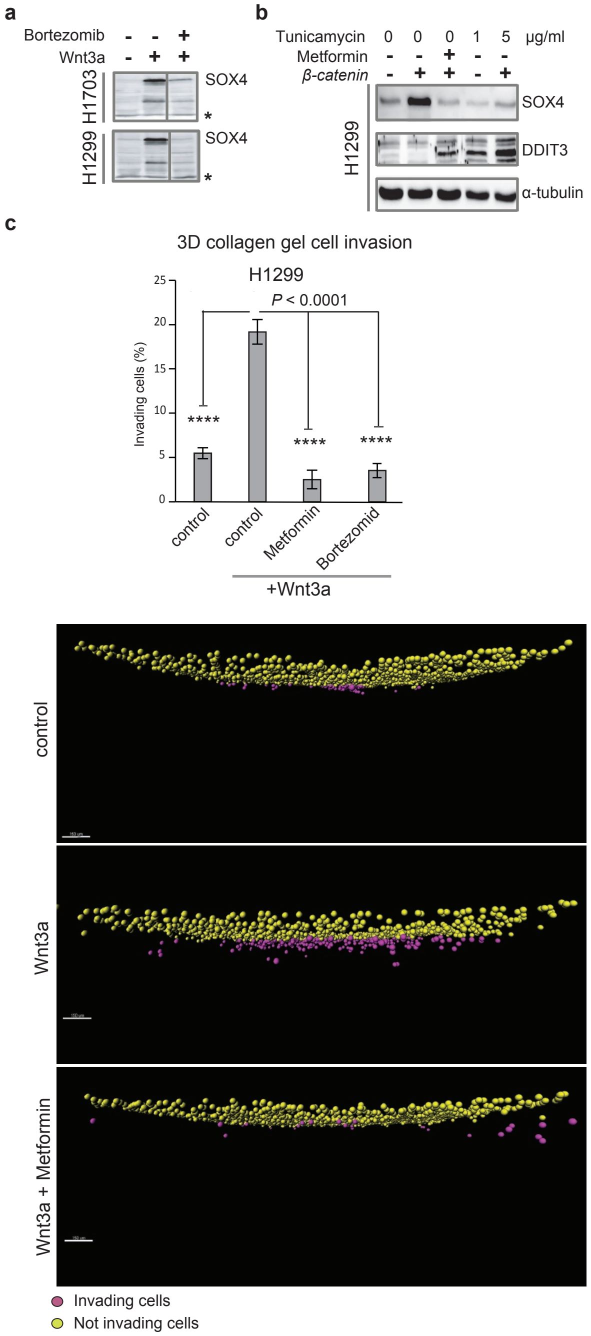
a
Bortezomib - - +
Wnt3a - + +
H1703
SOX4
*
H1299
SOX4
*
b
Tunicamycin 0 0 0 1 5 µg/ml
Metformin - - + - -
β-catenin - + + - +
H1299
SOX4
DDIT3
α-tubulin
c
3D collagen gel cell invasion
H1299
P < 0.0001
Invading cells (%)
25
20
15
10
5
0
****
****
****
control
control
Metformin
Bortezomid
+Wnt3a
control
Wnt3a
Wnt3a + Metformin
Invading cells
Not invading cells

Melnik *et al.,* Supplementary Figure 7

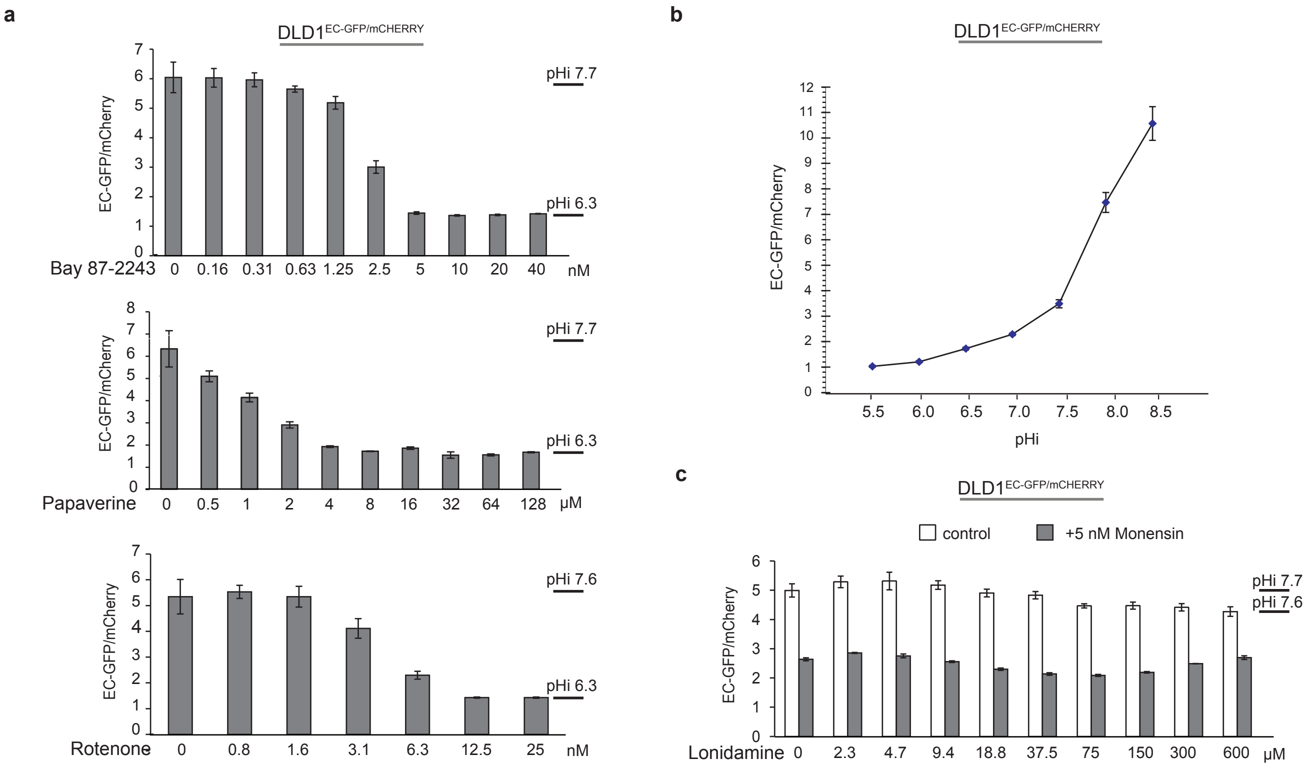

Melnik *et al.,* Supplementary Figure 8

a

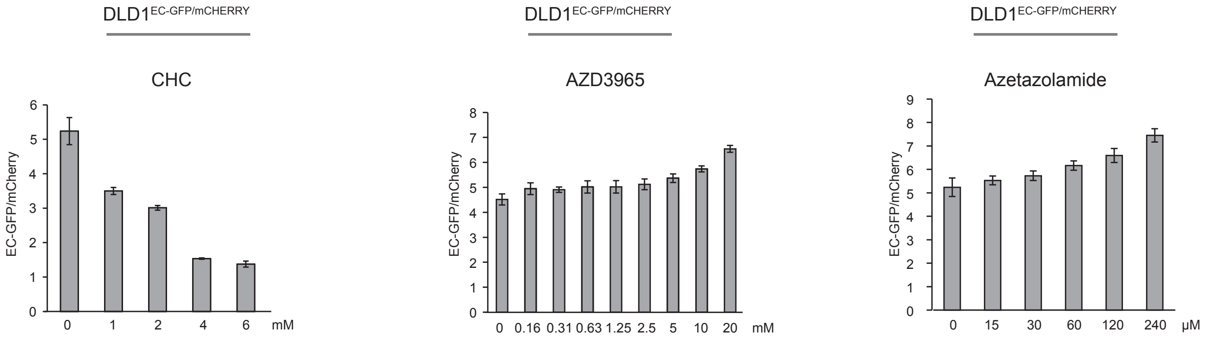


b

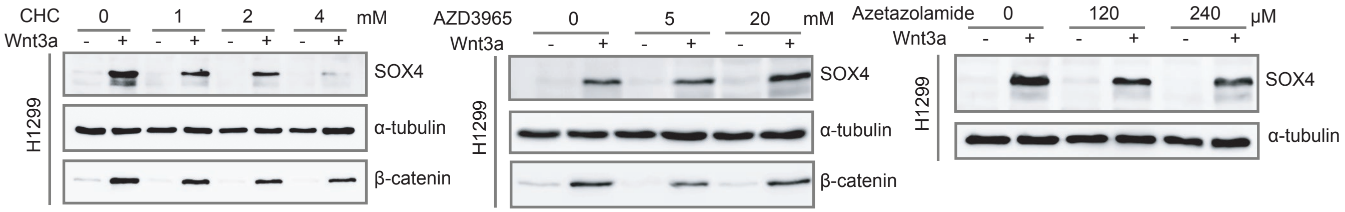

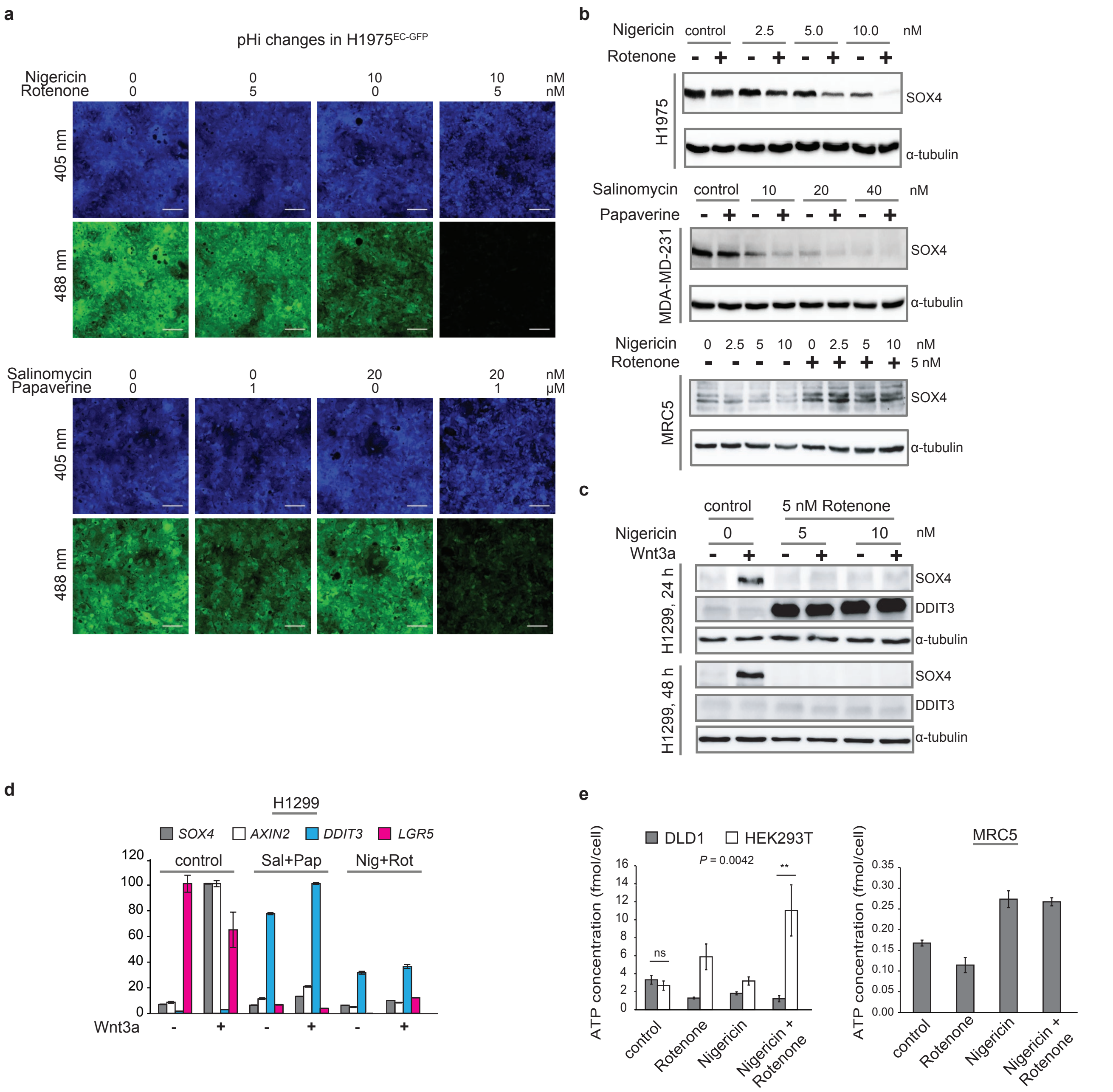
a
pHi changes in H1975EC-GFP
Nigericin 0 0 10 10 nM
Rotenone 0 5 0 5 nM
405 nm
488 nm
Salinomycin 0 0 20 20 nM
Papaverine 0 1 0 1 μM
405 nm
488 nm
b
Nigericin control 2.5 5.0 10.0 nM
Rotenone - + - + - + - +
H1975
SOX4
α-tubulin
Salinomycin control 10 20 40 nM
Papaverine - + - + - + - +
MDA-MD-231
SOX4
α-tubulin
Nigericin 0 2.5 5 10 0 2.5 5 10 nM
Rotenone - - - - + + + + 5 nM
MRC5
SOX4
α-tubulin
c
control 5 nM Rotenone
Nigericin 0 5 10 nM
Wnt3a - + - + - +
H1299, 24 h
SOX4
DDIT3
α-tubulin
H1299, 48 h
SOX4
DDIT3
α-tubulin
d
H1299
SOX4 AXIN2 DDIT3 LGR5
control Sal+Pap Nig+Rot
120 100 80 60 40 20 0
Wnt3a - + - + - +
e
DLD1 HEK293T
P = 0.0042
ns
**
ATP concentration (fmol/cell)
16 14 12 10 8 6 4 2 0
control Rotenone Nigericin Nigericin + Rotenone
MRC5
0.35 0.30 0.25 0.20 0.15 0.10 0.05 0.00
control Rotenone Nigericin Nigericin + Rotenone

**a**

**b**

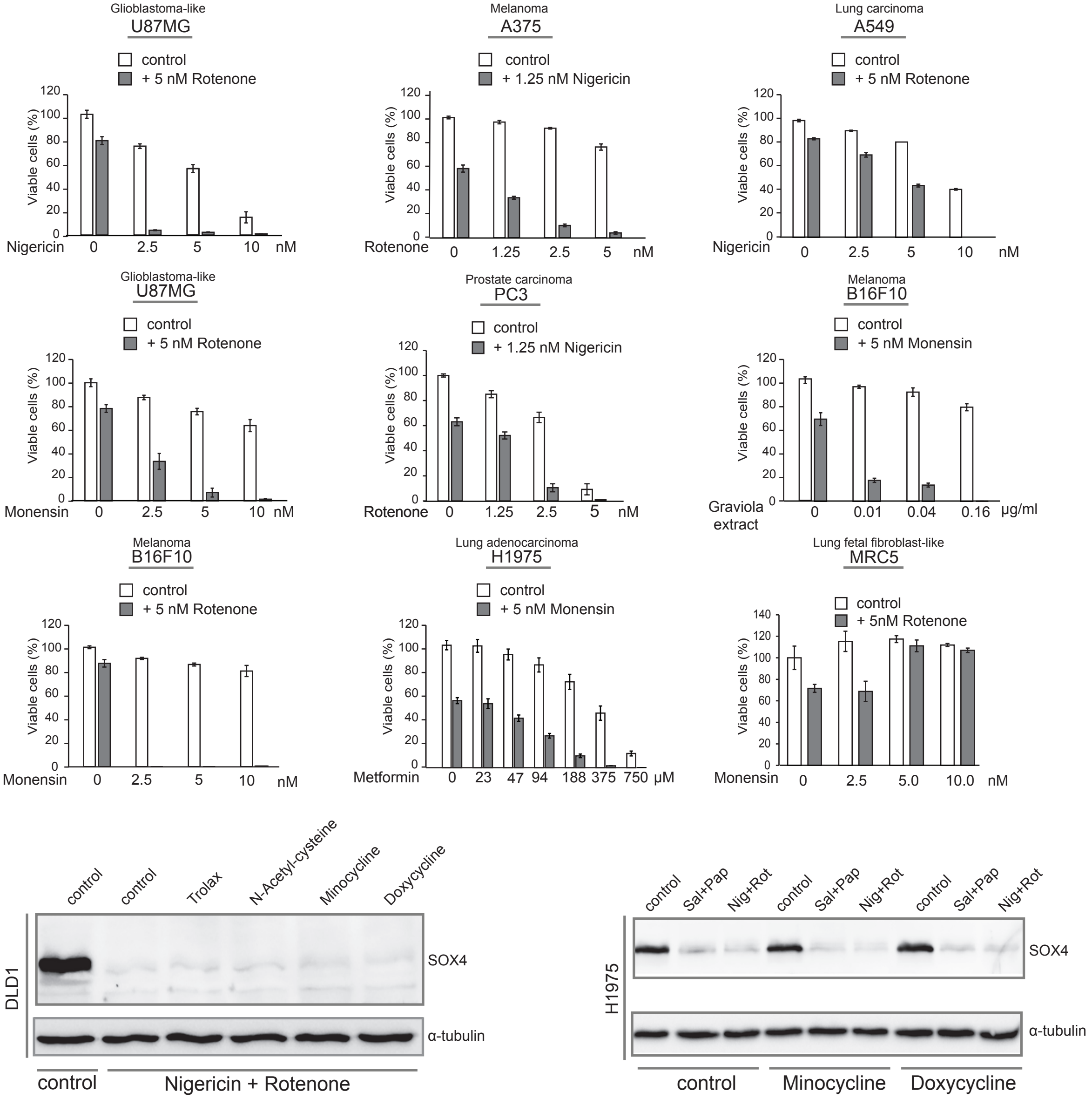
Glioblastoma-like
U87MG
control
+ 5 nM Rotenone
Viable cells (%)
Nigericin 0 2.5 5 10 nM
Melanoma
A375
control
+ 1.25 nM Nigericin
Rotenone 0 1.25 2.5 5 nM
Lung carcinoma
A549
control
+ 5 nM Rotenone
Nigericin 0 2.5 5 10 nM
Glioblastoma-like
U87MG
control
+ 5 nM Rotenone
Monensin 0 2.5 5 10 nM
Prostate carcinoma
PC3
control
+ 1.25 nM Nigericin
Rotenone 0 1.25 2.5 5 nM
Melanoma
B16F10
control
+ 5 nM Monensin
Graviola extract 0 0.01 0.04 0.16 μg/ml
Melanoma
B16F10
control
+ 5 nM Rotenone
Monensin 0 2.5 5 10 nM
Lung adenocarcinoma
H1975
control
+ 5 nM Monensin
Metformin 0 23 47 94 188 375 750 μM
Lung fetal fibroblast-like
MRC5
control
+ 5nM Rotenone
Monensin 0 2.5 5.0 10.0 nM
control
control
Trolax
N-Acetyl-cysteine
Minocycline
Doxycycline
DLD1
SOX4
α-tubulin
control
Nigericin + Rotenone
control
Sal+Pap
Nig+Rot
control
Sal+Pap
Nig+Rot
control
Sal+Pap
Nig+Rot
H1975
SOX4
α-tubulin
control
Minocycline
Doxycycline
